# Equatorial Pacific seawater *p*CO_2_ variability since the last glacial period

**DOI:** 10.1038/s41598-019-49739-0

**Published:** 2019-09-25

**Authors:** Kaoru Kubota, Yusuke Yokoyama, Tsuyoshi Ishikawa, Takuya Sagawa, Minoru Ikehara, Toshitsugu Yamazaki

**Affiliations:** 1 Kochi Institute for Core Sample Research, Japan Agency for Marine-Earth Science and Technology, Nankoku, Japan; 2 Atmosphere and Ocean Research Institute, The University of Tokyo, Kashiwa, Japan; 3 Institute of Science and Engineering, Kanazawa University, Kanazawa, Japan; 4 Center for Advanced Marine Core Research, Kochi University, Nankoku, Japan

**Keywords:** Palaeoceanography, Palaeoclimate

## Abstract

The ocean may have played a central role in the atmospheric *p*CO_2_ rise during the last deglaciation. However, evidence on where carbon was exchanged between the ocean and the atmosphere in this period is still lacking, hampering our understanding of global carbon cycle on glacial–interglacial timescales. Here we report a new surface seawater *p*CO_2_ reconstruction for the western equatorial Pacific Ocean based on boron isotope analysis—a seawater *p*CO_2_ proxy—using two species of near-surface dwelling foraminifera from the same marine sediment core. The results indicate that the region remained a modest CO_2_ sink throughout the last deglaciation.

## Introduction

During the last deglaciation (ca. 19.0–10.5 ka), the atmospheric partial pressure of carbon dioxide (*p*CO_2_) increased by approximately 80 μatm^1–3^. Lines of evidence suggest that the deep-ocean carbon reservoir played a central role in the rise in atmospheric CO_2_, since its carbon storage capacity is 60 times larger than that of the atmosphere^4–8^. However, evidence on where carbon was exchanged between the ocean and the atmosphere is still lacking, hampering our understanding of global carbon cycle on glacial–interglacial timescales.

**Figure 1:**
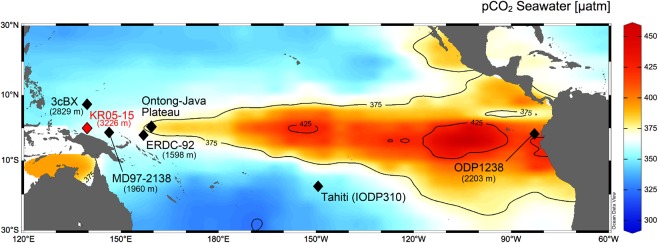
Sea surface *p*CO_2_ in the equatorial Pacific. Equatorial Pacific locations discussed in the text with sea surface *p*CO_2_ values for the reference year 2000 CE. Red and blue areas represent CO_2_ sources and sinks, respectively^42^. Diamonds indicate the locations of marine sediment cores and fossil corals mentioned in this study.

The boron isotope ratio (δ^11^B) of the skeletons of marine calcifying organisms such as corals and foraminifera can be used to infer where a CO_2_ source or sink existed, because the δ^11^B value of marine calcium carbonates is dependent on seawater pH, from which the *p*CO_2_ of the seawater can be reconstructed^9–12^. Investigating current oceanic source regions of CO_2_, such as the equatorial Pacific, is of great importance because such regions were likely sites of CO_2_ transfer from the deep-sea to the atmosphere in the past, as they are today (Fig. 1). In previous studies that measured δ^11^B in biogenic calcium carbonates, Martinez-Boti *et al*.^10^ (ODP Site 1238, Fig. 1) and Kubota *et al*.^11^ (IODP Exp. 310) revealed that central and eastern parts of the equatorial Pacific (CEP and EEP, respectively) acted as CO_2_ sources during the last deglaciation, suggesting that the equatorial Pacific ocean partly contributed to the rise in atmospheric CO_2_. However, some controversial results have been obtained in a marine sediment record from the western equatorial Pacific (WEP)^12^. Based on δ^11^B measurements of the planktonic foraminifera *Torilobatus sacculifer* in a marine sediment core (ERDC-92, Fig. 1), Palmer and Pearson^12^ reported that the surface layer of the WEP was a CO_2_ source during the last deglaciation, which suggested a potential contribution of this region to the deglacial rise in atmospheric CO_2_. However, their reported ranges of δ^11^B values differ from generally accepted values for *T. sacculifer* on glacial-interglacial timescales^9,10,13–16^ by as much as 5‰, which is likely due to positive bias, especially when analyzing foraminifera shell using negative thermal ionization mass spectrometry (N-TIMS)^15,17–19^. Considering that glacial–interglacial changes in δ^11^B measured in foraminifera are as small as approximately 2‰, this large discrepancy cannot be overlooked. The δ^11^B measurement of biogenic calcium carbonate using N-TIMS method has started since the end of 1980s (ref.^20^), and there has been a significant development in analytical procedure^15–19^. However, it still suffers from mass fractionation during ionization of the sample, resulting in a larger analytical uncertainty as well as less accuracy, especially when foraminifera sample is analyzed^17–19^. The δ^11^B measurement using multi-collector inductively coupled plasma mass spectrometry (MC-ICPMS) has started since the beginning of 2000s (refs^21,22^), which has become more widely-accepted and conventional ways to determine δ^11^B values, providing more precise and accurate values (e.g., refs^9–11,13–19,23,24^). Therefore, the refinement of seawater *p*CO_2_ records derived from the δ^11^B of planktonic foraminifera from the WEP, determined by MC-ICPMS method, is of great importance for understanding the role of the equatorial Pacific in the deglacial rise in atmospheric CO_2_. The WEP drives the world’s most intense atmospheric convection, being regarded as a heat engine of earth climate system^25^, thus to understand past oceanographic variability is of great importance not only for carbon cycle but also for climatology. For this purpose, we chose a marine sediment core recovered from the West Caroline Basin (KR05-15 PC01, 0.10°S 139.58°E, 3226 m below sea level, Fig. 1)^26^. A chronology of core KR05-15 PC01 was constructed based on 17 radiocarbon dates and deterministic age-depth modeling^27^, as well as oxygen isotope measurements of benthic foraminifera shells (Supplementary Fig. 1).

## Boron Isotopes and Seawater CO_2_ System Reconstruction

In this study, we analyzed two species of surface-dwelling planktonic foraminifera—*Globigerinoides ruber* (white) and *T. sacculifer*. We collected foraminifera shells from size fractions generally used in paleoceanographic studies, namely, 300–355 μm and 500–855 μm, for *G. ruber* and *T. sacculifer* (with a sac-like final chamber), respectively^9,10,13–16,24^. To calculate *p*CO_2_, it is necessary to measure not only δ^11^B but also the Mg/Ca ratio (a temperature proxy) and oxygen isotope ratio (δ^18^O, a salinity proxy). Thus, we established a way to obtain these three values from a single sample, which requires 3–7 mg of foraminifera shells, from which shells are removed that potentially contain fragments of shells from other calcifying organisms and siliciclastic grains (Supplementary Figs S2–S7).

**Figure 2:**
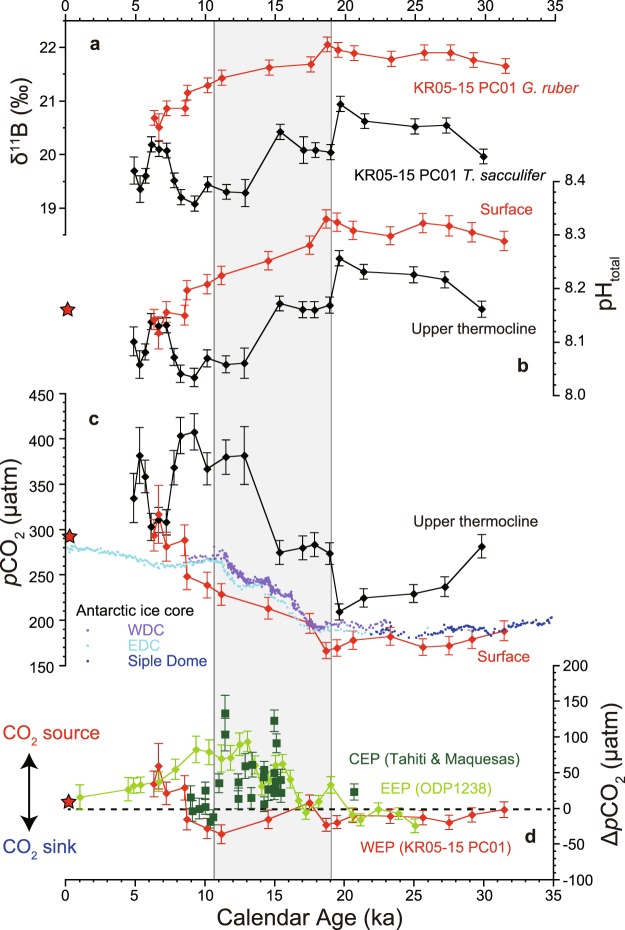
Deglacial changes of seawater CO_2_ system. Deglacial δ^11^B, pH, *p*CO_2_, and Δ*p*CO_2_ (seawater *p*CO_2_ minus atmospheric *p*CO_2_) variability in the equatorial Pacific. (**a**) δ^11^B values of *G. ruber* (red) and *T. sacculifer* (black) from core KR05-15 PC01 with 2σ analytical uncertainty. (**b**) pH (in total hydrogen scale) of surface and upper-thermocline seawater reconstructed from δ^11^B. **(c)** Reconstructed *p*CO_2_ of surface and upper-thermocline seawater and atmospheric *p*CO_2_ reconstructed from Antarctic ice cores (West Antarctic Ice Sheet Divide ice core, WDC; Siple Dome; EPICA Dome C, EDC)^1–3^. (**d**) Reconstructed *p*CO_2_ of surface seawater in the WEP (red, this study), EEP (light green)^10^, and CEP (dark green)^11,23^. Error bars of pH, *p*CO_2_, and Δ*p*CO_2_ were calculated using a Monte Carlo approach. The shaded area indicates the time period of the last deglaciation.

**Figure 3:**
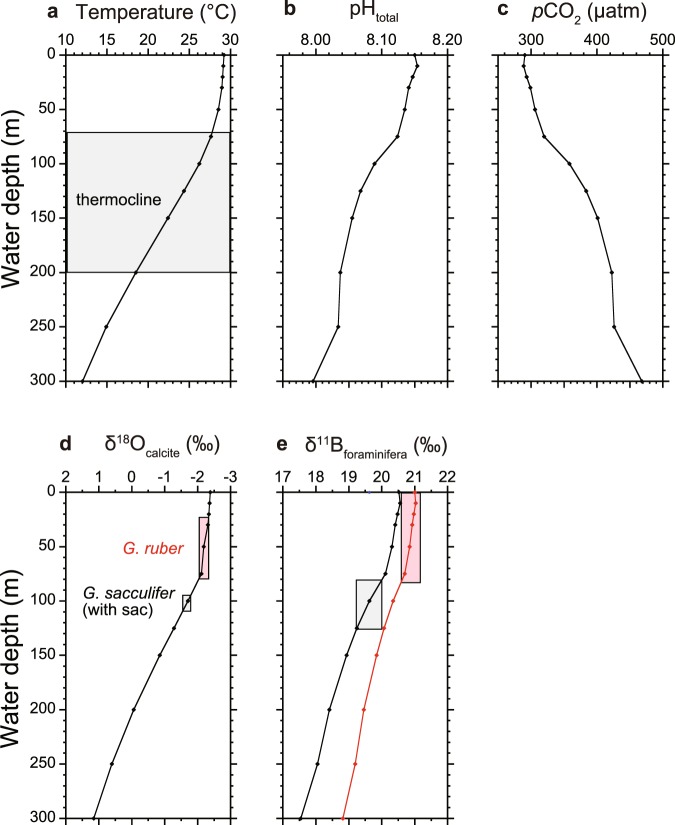
Depth profile of seawater CO_2_ system and calcification depth estimation of planktonic foraminifera. Depth profile of seawater properties and calcification depth estimation of *G. ruber*, and *T. sacculifer* (with sac-like final chamber), in the WEP. (**a**) Depth profile of water temperature^74^. The shaded area indicates thermocline depth. (**b**) Seawater pH and (**c**) seawater *p*CO_2_ calculated using pre-industrial dissolved inorganic carbon and total alkalinity^43^. (**d**) δ^18^O and (**e**) δ^11^B values estimated using water temperature, salinity, seawater pH, and species-specific δ^11^B–pH calibration equations.

The δ^11^B values obtained in this study lie within the range obtained by Henehan *et al*.^13^ for both *G. ruber* and *T. sacculifer*, and are also consistent with the reported δ^11^B values from the core-top material of marine sediment collected from the Ontong Java Plateau in the WEP^14^ (Fig. 1, Supplementary Fig. S7). Both foraminifera records show steady δ^11^B values during the last glacial period (31–20 ka) (Fig. 2a), and both then show gradual decreases in δ^11^B, starting at the beginning of the last deglaciation, with the onset of the decrease in *T. sacculifer* preceding that in *G. ruber* by approximately 3 ky. Before 8 ka, the δ^11^B values of *T. sacculifer* are always lower than those of *G. ruber*, with a difference of 1.0–1.5‰. We estimate the calcification depth of *G. ruber* and *T. sacculifer* from theoretically predicted δ^18^O and δ^11^B values using data spanning the Holocene (4.0–10.5 ka). Since water temperature and pH (and *p*CO_2_) vary with water depth (Fig. 3a–c), the measured δ^18^O and δ^11^B values of foraminifera shells can be used to estimate the mean water depth at which the calcite shells precipitated. The theoretical δ^18^O depth profile of foraminifera calcite is calculated from temperature and seawater δ^18^O values. For the calculation of theoretical δ^11^B depth profile of both species, seawater pH is used, in which a decrease of pH due to anthropogenic CO_2_ incorporation since the Industrial Revolution is considered. The estimates of calcification depth based on different isotopes showed good agreement (Fig. 3d,e). Estimated calcification depths were 0–75 m (within the mixed layer) for *G. ruber*, and 75–125 m (below the mixed layer; upper thermocline) for *T. sacculifer* (Fig. 3), which is consistent with independent estimates from marine sediment cores collected in the WEP^28–30^. In the present study, *T. sacculifer* shells recorded colder, lower pH, and higher *p*CO_2_ water compared with *G. ruber* shells (Figs 2 and 3).

## Shell Dissolution Effect

Because our core was obtained from water below the calcite saturation horizon depth (the saturation state with respect to calcite, Ω_calcite_, is 0.87), the bottom water is corrosive to calcite. We investigated the potential influence of dissolution of the *G. ruber* and *T. sacculifer* foraminifera shells on the δ^18^O and δ^11^B values, and thus on the mean calcification depth estimates and *p*CO_2_ reconstructions.

For *G. ruber*, both δ^18^O and δ^11^B of the shell is insensitive to dissolution^14,31–33^ (Supplementary Text). This is also supported by the fact that the δ^18^O variation of *G. ruber* in core KR05-15 PC01 carries an identical environmental signal to those collected from a nearby sediment core that has an excellent preservation of foraminifera shells (MD97-2138; Fig. 1, Supplementary Fig. S5)^29^. For *T. sacculifer*, on the other hand, shell dissolution effects on its geochemistry is not as straightforward as *G. ruber*, which is because of a presence of the sac-like final chamber. The sac-like final chamber and a gametogenic calcite coating are precipitated during the last life stage of this animal^30,33–37^. During the reproduction, they migrate into the thermocline depth and release gametes, which is governed by lunar cycles^30,34,35,37^. The mean calcification depth of *T. sacculifer* in the WEP is deeper than global mean, likely due to thick mixed layer and chlorophyll maximum in the thermocline depth^30^. For example, Rebotim *et al*.^37^ estimate using plankton tow that the mean calcification depth of *T. sacculifer* is shallower in the subtropical North Atlantic, ~60 m. Furthermore, the sac is likely more resistant to corrosive bottom water, thus the measurement of *T. sacculifer* with sac leads to a lower temperature (lower δ^18^O) and lower pH (lower δ^11^B) reconstruction^33,38^. However, two observation support that the presence of the sac-like final chamber does not alter either the mean calcification depth estimate of *T. sacculifer* or the *p*CO_2_ estimate of upper-thermocline seawater in the WEP (see Supplementary Text, for more thorough discussion). First, *T. sacculifer* with a sac-like final chamber in core KR05-15 PC01 carries an identical environmental signal (that is Mg/Ca and δ^18^O) to those without sac collected from a nearby sediment core that has an excellent preservation of foraminifera shells (MD97-2138; Fig. 1, Supplementary Fig. S6)^29^. Second, δ^11^B values of *G. sacculifer* from core KR05-15 PC01 are consistent with those from core-top material from the Ontong Java Plateau which is not affected by dissolution^14^ (Fig. 1, Supplementary Fig. S7). Thus we conclude that relatively deeper calcification depth is due to location-specific habitat depth preferences of *T. sacculifer*, not due to dissolution effects. The reason of this is likely that the modest sedimentation rate of KR05-15 PC01 (4.5 cm/ky) enabled the fine preservation of foraminifera shells, even though the bottom water was undersaturated. It is also noteworthy that large *T. sacculifer* individuals are less influenced by dissolution, because of a relative proportion of sac versus other chambers are smaller in large individuals^38^. It is likely that dissolution effects is negligible through the record, because there are little variation in saturation state in the Pacific deep-water in glacial-interglacial timescales^39^. We also found that the selection of different Mg/Ca–water temperature relationships and consideration of an effect of partial dissolution of *G. ruber* shell^28,31,40^ has a negligible effect on the calculation of surface seawater *p*CO_2_ (Supplementary Text, Supplementary Fig. S8). Similarly we found that pH effect on Mg/Ca–water temperature relationships and different total alkalinity estimation scenario since the last glacial period has a negligible effect on the calculation of seawater *p*CO_2_ (Supplementary Text, Supplementary Figs S9–S11).

## Surface Seawater *p*CO_2_

Today (at the reference year 2000 CE), the surface seawater *p*CO_2_ of the WEP has a similar value (360 μatm) to the atmospheric value (370 μatm) (Fig. 1)^41,42^. A CO_2_ neutral condition (seawater *p*CO_2_ is close to atmospheric *p*CO_2_) is also likely to have existed in pre-industrial times when the atmospheric *p*CO_2_ value was 280 μatm (ref.^3^). The *p*CO_2_ value of the surface seawater is calculated as 290 μatm (10 μatm higher than the atmospheric value) based on recent instrumental seawater CO_2_ system observations made using research vessels and theoretical calculations considering anthropogenic CO_2_ incorporation since pre-industrial times^43^ (Figs 2c,d and 3c). From the last glacial period to the early Holocene, reconstructed seawater *p*CO_2_ values of mixed-layer water obtained using *G. ruber* show variation similar to that of atmospheric values, suggesting that the oceanic surface layer in the WEP remained in a CO_2_ neutral condition (Fig. 2c,d). During the mid- Holocene (6.4–8.6 ka), the WEP became a modest source of CO_2_ (on average +36 μatm higher than the atmospheric *p*CO_2_), which is consistent with modern oceanographic observations (Fig. 2b,c), suggesting that *G. ruber* shells are a reliable recorder of surface seawater CO_2_ chemistry. Considering the estimation error of Δ*p*CO_2_ (the difference between seawater *p*CO_2_ and atmospheric *p*CO_2_) from the δ^11^B value of *G. ruber*, Δ*p*CO_2_ was near zero through the last deglaciation (Fig. 2d), showing that there is no evidence of CO_2_ release from the WEP during this period. This result is in clear contrast with a previous finding by Palmer and Pearson^12^ that the WEP was a CO_2_ source region. The reason for this is most likely that *T. sacculifer* (the species used by Palmer and Pearson^12^) record pH and *p*CO_2_ values of the upper thermocline, not of the surface water in the WEP. This point is also of great importance for the reconstruction of atmospheric CO_2_ using δ^11^B analysis of *T. sacculifer* shells for the deep past, beyond the limit of Antarctic ice cores (approximately, 800 ka)^9,15,16,24^, as a deeper calcification depth than the one we assume may result in the overestimation of atmospheric *p*CO_2_. We note that previous reconstruction using δ^11^B of *T. sacculife*r in the marine sediment core collected from the equatorial Atlantic is irrelevant to this issue (see the recent compilation by Dyez *et al*.^15^), because their calcification depth is confirmed to lie within mixed layer.

## A Linkage Between High and Low Latitude Ocean

**Figure 4:**
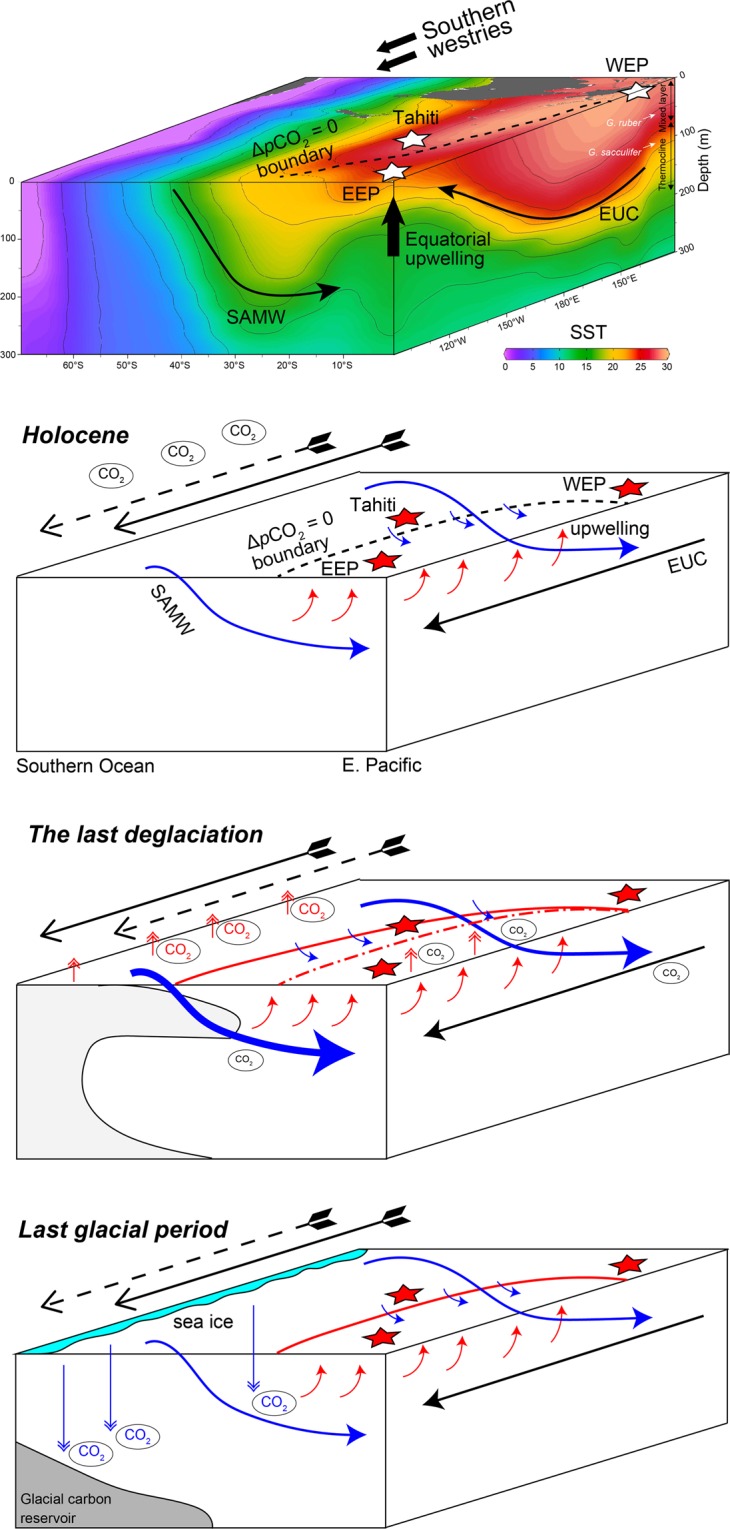
Schematics of deglacial marine carbon cycles. Schematics of carbon cycles and climatology in the South Pacific Ocean for three time periods: the last glacial period, the last deglaciation, and the Holocene.

During the last glacial period, an efficient biological pump due to iron fertilization and increased stratification of the Southern Ocean due to sea ice expansion around Antarctica resulted in more efficient carbon storage in the deep sea and a drop in the atmospheric *p*CO_2_ level^7,8,44^ (Fig. 4). During the last deglaciation, upwelling in the Southern Ocean was enhanced, and carbon was returned to the atmosphere^8,45,46^. This event was likely accompanied by a stronger connection between the Southern Ocean and surface and thermocline depth of the low-latitude Pacific, which is known as “geochemical tunneling” (Fig. 4). A large variety of geochemical tracers have suggested greater enhancement of water transport due to Ekman pumping by the southward migration of southern westerlies^7^, more aged water mass incorporation into the intermediate water^6,11,45–47^, and more pronounced primary productivity in the Southern Ocean^8,48^. Higher surface *p*CO_2_ conditions during the last deglaciation reported for the EEP^10^ and the CEP (Tahiti)^11,23^ (Fig. 3) were likely a result of these phenomena, as such a water mass was likely accompanied by higher *p*CO_2_ water originating from the glacial deep-sea carbon reservoir (Fig. 4). The fact that no high-*p*CO_2_ water was observed in the mixed layer of the WEP suggests that the high*-p*CO_2_ region expanded southward but not westward (Fig. 4). Our results suggest that throughout deglaciation all of the nutrients/CO_2_ upwelled in the EEP were fully utilized by the time these surface waters arrived in the WEP. Additionally, the abovementioned 3-ky precedence of the increase in upper thermocline *p*CO_2_ (that is decrease in δ^11^B) in the WEP at the beginning of the last deglaciation (recorded in *T. sacculifer* shells) relative to the increase in surface *p*CO_2_ (recorded in *G. ruber* shells) was likely a result of an early arrival of high *p*CO_2_ water to the upper thermocline at approximately 19 ka. A possible source of such water mass is the Southern Ocean, especially via water transport via the Subantarctic Mode Water (SAMW) and Equatorial Undercurrent (Fig. 4). Considering that atmospheric CO_2_ began to increase only after about 17.5 ka, as determined from Antarctic ice core records (Fig. 2d), a stronger upwelling condition in the Southern Ocean and subsequent incorporation of high-*p*CO_2_ water into the SAMW might have started at 19 ka, although it was not enough to fuel atmospheric CO_2_. This idea is partly supported by the EEP surface ocean records at 19 ka that show significantly higher *p*CO_2_ than in the atmosphere (i.e., positive Δ*p*CO_2_)^10^ (Fig. 3). Another important observation is that surface ocean *p*CO_2_ in the EEP and the CEP and subsurface *p*CO_2_ in the WEP was remained high although atmospheric *p*CO_2_ did not increase during the Bølling–Allerød warm interval (B/A; 14.6–12.9 ka). Surface *p*CO_2_ reconstruction using δ^11^B of planktonic foraminifera, *Neogloboquadrina pachyderma*, collected from marine sediment core in the North Pacific (51.27°N, 167.73°E)^49^ shows similar variation to our subsurface *p*CO_2_ record, which may provide a hint to understand the mechanism behind, because there is another path that transport nutrient and carbon from the North Pacific to the equatorial Pacific via North Pacific Intermediate Water (NPIW)^50^. It is noteworthy that at the onset of the B/A interval, a collapse of NPIW formation is suggested^49^, which occurred at the same time of a reduction of the southern-sourced water contribution in the thermocline depth of the equatorial Pacific^5^. A hypothesis that *p*CO_2_ variability of thermocline in the equatorial Pacific is driven by both north and south high-latitude ocean processes merits testing by obtaining more high-resolution records from wider spatial coverage. In addition, other important research topics include investigating lower-thermocline conditions by utilizing thermocline-dwelling foraminifera shells (e.g., refs^5,28^) and investigating seawater *p*CO_2_ conditions during other glacial terminations, such as the penultimate deglaciation. For these purposes, the boron isotope proxy is a promising tool to create a *p*CO_2_ map in order to better understand the redistribution of CO_2_ between seawater and the atmosphere during glacial–interglacial cycles. Given a large surface area and its connectivity to the deep ocean, the Southern Ocean most likely had a central role in deglacial CO_2_ release into the atmosphere, but relative importance of CO_2_ flux from the Southern Ocean and that from other oceanic regions including the equatorial Pacific remains unquantified.

## Materials and Methods

### Geochemical analyses

We analyzed a marine sediment core obtained from the West Caroline Basin (0.10°S 139.58°E) in 2005 by R/V Kairei. A 15-m-long sediment core that covers the last 400 ka was obtained using a piston coring system (core KR05-15 PC01)^26^. The top 1.5 m of the core was used for this study, as it covers the time period since the last glacial period. Sediment lithologies are foraminifera-rich clay (brown) for the top 30 cm and clay with foraminifera (greenish gray) from a depth of 30 cm to 150 cm. The archive half was cut in 2 cm intervals, and the sediment samples were gently washed under running water over a 63 μm sieve and dried at 60 °C in an oven. The sieved materials were further divided into size fractions of 63–255, 255–300, 300–355, 355–425, 425–500, and 500–850 μm, and stored in plastic vials.

The age model of the sediment core was established by 17 radiocarbon dates measured by accelerator mass spectrometry (AMS). If enough calcium carbonate materials were available, shells of mono-specific planktonic foraminifera (*T. sacculifer*) were used; otherwise, shells of mixed planktonic foraminifera were used (Supplementary Table S1). Seven samples were submitted as carbonate to the National Ocean Sciences Accelerator Mass Spectrometry Facility (NOSAMS), Woods Hole Oceanographic Institution (WHOI), and the remaining samples were analyzed in the Atmosphere and Ocean Research Institute (AORI), University of Tokyo. Three of the 20 samples were omitted as they showed anomalous values compared with neighboring data. Conventional ^14^C ages were converted into calendar age before 1950 CE using a Marine13 calibration curve^51^ with a local marine ^14^C reservoir age of 40 ± 21 years^52,53^ (Supplementary Fig. S1).

More than two shells of *Uvigerina* spp. were hand-picked under a microscope from the sediment samples from size fractions of 300–355 and 355–425 μm. Shells of *G. ruber* (white, s.s./s.l.) and *T. sacculifer* (with a sac-like final chamber) were hand-picked under microscope from size fractions of 300–355 and 500–850 μm, respectively. All samples were weighed, and if the sample amount was insufficient for the subsequent geochemical analysis, some continuous samples were mixed (at most eight samples).

Oxygen isotopes of benthic foraminifera *Uvigerina* spp. were measured using an isotope-ratio mass spectrometer (Isoprime) with an automated carbonate reaction system (Multiprep) installed at Kochi Core Center (KCC), Japan. The values of δ^18^O are reported with respect to the Vienna Pee Dee Belemnite standard using standard delta notation in permil^54^. Analytical precision for long-term measurement of the calcite standard (IAEA603) was better than 0.1‰. A drift correction for the sample δ^18^O values was performed, as the long-tem drift of the measured IAEA603 values of the standards during the measurement period between June 2017 and June 2018 was observed to be 0.12–0.32‰ isotopically heavier than the certified value of −2.37‰ (IAEA Reference Products; available at http://nucleus.iaea.org/rpst/ReferenceProducts/ReferenceMaterials/Stable_Isotopes/index.htm).

A technique for measuring Mg/Ca, δ^18^O, and δ^11^B from the same planktonic foraminifera sample was established in this study (Supplementary Fig. S2). The foraminifera cleaning methodology was after Baker *et al*.^55^. Weighed samples were crushed gently between two acrylic slides until the insides of all the chambers were visible under stereoscopic microscope. Samples were subsequently transferred to 1.5 mL Eppendorf tubes. Samples were sonicated five times with Milli-Q water and twice with methanol. The supernatant containing suspended fine grains was removed immediately from the sample after ultrasonication. To remove organic matter by oxidation, 0.1 N NaOH buffered 30% H_2_O_2_ was added and heated at 80 °C for 10 min. Subsequent reductive steps were skipped, as they do not have a discernible effect on δ^11^B measurement^33,56^.

Several hundred micrograms of samples were divided for δ^18^O measurements using the Isoprime spectrometer (Supplementary Fig. S2). The methodology of δ^18^O measurements was the same as above. The remaining samples were transferred to acid-cleaned Teflon vials. After weak acid-leaching of any adhesive materials attached to calcium carbonate, 1–2 mL 0.1 M HCl was added to the samples until complete dissolution, together with 5 μL of 1% mannitol solution. A total of 30 μL of sample was taken from the solution and diluted with 5 mL of 0.15 M HNO_3_ with internal standards (50 ppb Be and 100 ppb Sc, Y, and In). First, Ca concentration was measured using a quadrupole inductively coupled plasma mass spectrometer (Q-ICPMS; Agilent7700, Agilent Technology) installed at KCC (Supplementary Fig. S2). Then, 30 μL of sample was taken again from the solution and diluted to make the Ca concentration of each sample 10 ppm (matrix matching). Matrix matching is essential for the precise determination of trace element/Ca ratios^28,57^. Standard solution (200 ppb of Mg and Sr, 50 ppb of B, Al, Mn, Rb, and Ba) was prepared by diluting commercially available 1000 μg/g single element standards with the abovementioned 0.15 M HNO_3_ containing internal standards. Elemental ratios of samples were determined using the Agilent7700 instrument, with a machine drift correction. A calcium carbonate standard JCp-1, provided by the Geological Survey of Japan, was repeatedly analyzed to calculate the precision of the trace element/Ca ratio measurements. The precision of Mg/Ca analysis was 3.4% (1σ).

Boron isotopic measurements were conducted following the methodology of Tanimizu *et al*.^58^. Briefly, the boron was purified using cation- and anion-exchange resin columns, and the samples were dissolved with an acid mixture composed of 0.15 M HNO_3_, 0.05 M HF, and 0.1% mannitol to obtain a solution of 20–50 ppb B. Li standard (^7^Li/^6^Li isotopic reference) was added to each sample to correct a mass discrimination. δ^11^B values were determined with a multi-collector inductively coupled plasma mass spectrometer (MC-ICPMS) (Neptune, Thermo Finnigan) installed at KCC, against the isotopic reference NIST-SRM 951, using a standard-sample bracketing technique under wet plasma conditions. Since July 2017, a jet sampling cone instead of a normal sampling cone has been used with an X skimmer cone, which significantly increased beam intensity for both carbonate standard JCp-1 and seawater standard AE122 (Supplementary Fig. S3). δ^11^B values of JCp-1 were determined with different boron concentrations (10, 20, 30, 40, 50, and 75 ppb), which yielded consistent values with a mean of 24.38 ± 0.28‰ (2σ, n = 37) only if ^11^B beam intensity was greater than 0.2 V (Supplementary Fig. S3); in the case of beam intensity of less than 0.2 V, analytical error increased to ±0.5‰ (2σ, n = 10). δ^11^B values of AE122 were determined with a uniform concentration of 75 ppb, which yielded a mean value of 39.48 ± 0.24‰ (2σ, n = 14). As with JCp-1, the measurement of AE122 using a jet sampling cone resulted in higher beam intensity than measurement using a normal cone, and there were no discernible differences in δ^11^B between selections of sampling cones (Supplementary Fig. S3). δ^11^B values of both JCp-1 and AE122 were within the previously published 2σ range of data compiled from different laboratories by Foster *et al*.^18^ and Foster *et al*.^59^. The analytical error of JCp-1 (±0.14‰, 1σ) was taken as the analytical errors of the foraminifera samples, except when ^11^B beam intensity was less than 0.2 V, in which case the larger error (±0.25‰, 1σ) was employed.

To assess the reproducibility of the methodology, Mg/Ca, δ^18^O, and δ^11^B of *T. sacculifer* samples in the Holocene section were measured 2–4 times, as they were abundant in this interval. The results of the Mg/Ca, δ^18^O, and δ^11^B replicates showed good agreement within the estimated analytical errors (Supplementary Fig. S4), supporting the reproducibility of this methodology.

### Age model

To obtain age-depth model of the sediment core KR05-15 PC01 in 1 cm intervals from 17 radiocarbon dates we used a recently published age-depth modeling tool “Undatable” (ref.^27^), which uses a deterministic approach with a positive sediment accumulation rate (Supplementary Fig. S1). We confirmed the robustness of the age model by comparing the δ^18^O values of *Uvigerina* spp. from core KR05-15 PC01 with a benthic δ^18^O record for Pacific deep-sea sediment^60^ (Supplementary Fig. S1). The values are in good agreement in the glacial period (30–18 ka), but there is an offset thereafter (maximum difference of 0.7‰). Although the absolute δ^18^O values are different, the shapes of the “up-down” patterns during 10–7 ka show excellent agreement. We infer that the difference is likely due to a regional difference in bottom-water temperature.

### Data screening

Some δ^11^B data from planktonic foraminifera that showed anomalously low values (1–2‰) were discarded from the calculation of *p*CO_2_. Such data were accompanied by higher Mg/Ca ratios (by more than 0.3 mmol/mol) as well as high Al counts (typically more than 50,000 cps), suggesting that boron was contaminated by silicate in the sediment^55,61^. Barker *et al*.^55^ investigated how the Mg/Ca ratio of foraminifera shells changes according to physical and chemical cleaning steps, and clearly showed that high Mg/Ca ratios result from insufficient removal of silicate (which is a major component of marine sediment). Similarly, without cleaning, the Al/Ca ratio varies in concert with the Mg/Ca ratio of foraminifera^55^, suggesting that Mg/Ca and Al content are useful to identify contaminated samples. As foraminifera shells were dissolved in 0.1 M HCl, excess boron likely originated from some material that is reactive with HCl. Ishikawa and Nakamura^61^ reported that the δ^11^B value of the HCl-soluble fraction of subtropical North Pacific marine sediment (nanno oose; DSDP313 1–5; 20°N 170°W) was 8.0‰ with a significant boron abundance (15 ppm), which is much lower than δ^11^B values of *G. ruber* (20–22‰) and *T. sacculifer* (19–21‰). Thus, we infer that the anomalously low foraminiferal δ^11^B values observed in this study can be explained by contamination by boron that was not removed sufficiently during the cleaning steps.

### Calculation of pH and *p*CO_2_

Water temperature and salinity need to be known for the calculation of seawater pH from the measured δ^11^B of foraminifera shells. Mg/Ca ratios of foraminifera shells were used for the calculation. The Mg/Ca–T equation of Sagawa *et al*.^28^ was used, as this equation was established by using a marine sediment core collected from a similar depth and location in the WEP (3cBX, 8.02°N, 139.64°E, 2829 m, Fig. 1).

$${\rm{T}}=1/0.077\,\ast \,\mathrm{ln}\,({\rm{Mg}}/{\rm{Ca}}/0.455)$$

where T is temperature in degrees Celsius.

Seawater δ^18^O (δ^18^O_SW_) was used to calculate salinity, as there is a linear relationship between salinity and δ^18^O_SW_. The δ^18^O of foraminifera calcite is a function of temperature and δ^18^O_SW_ (ref.^62^), so δ^18^O_SW_ can be calculated from δ^18^O and temperature (from Mg/Ca).

$${10}^{3}\,\ast \,\mathrm{ln}\,\alpha =18.03\,\ast \,{10}^{3}/({\rm{T}}+273.13)-32.42$$

or

$${\rm{T}}=16.1-4.64\,\ast \,{({\rm{\delta }}}^{18}{{\rm{O}}}_{{\rm{calcite}}}-{{\rm{\delta }}}^{18}{{\rm{O}}}_{{\rm{SW}}})+0.09\,\ast \,{({{\rm{\delta }}}^{18}{{\rm{O}}}_{{\rm{calcite}}}-{{\rm{\delta }}}^{18}{{\rm{O}}}_{{\rm{SW}}})}^{2}$$

Here, α is the fractionation factor and T is temperature in degrees Celsius (after ref.^63^). However, in glacial–interglacial timescales, past δ^18^O_SW_ changes need to be further considered for the calculation. Since the last glacial period, enormous volumes of freshwater with isotopically lighter δ^18^O values previously stored in continental ice sheets have entered the ocean, making global seawater δ^18^O lighter by ~1‰ (ice volume effect); as an additional consequence, sea level rose by ~135 m. As ice-derived δ^18^O_SW_ change has a linear relationship with global sea level, we estimated the ice volume effect from the reconstructed sea level curve of Yokoyama *et al*.^64^. We assume that a sea level change of 135 m causes a 1‰ change in δ^18^O_SW_ (ref.^65^). The ice volume-corrected δ^18^O_SW_ values were subsequently used for the calculation of salinity using the reported equation of Schmidt^66^:

$${\rm{S}}=0.422\ast {\delta }^{18}{{\rm{O}}}_{{\rm{S}}{\rm{W}}({\rm{i}}{\rm{c}}{\rm{e}}-{\rm{v}}{\rm{o}}{\rm{l}}{\rm{u}}{\rm{m}}{\rm{e}}-{\rm{c}}{\rm{o}}{\rm{r}}{\rm{r}}{\rm{e}}{\rm{c}}{\rm{t}}{\rm{e}}{\rm{d}})}-14.379$$

where S is salinity. There are known offsets between the theoretically expected δ^11^B of borate ions in the seawater (δ^11^B_borate_) and the measured δ^11^B of foraminifera calcite (δ^11^B_borate_), and these relationships differ among species (e.g., ref.^13^). For the calculation of seawater pH, the empirical relationships of Marinez-Boti *et al*.^67^ and Henehan *et al*.^23^ were used for *G. ruber* and *T. sacculifer*, respectively. Finally, seawater pH was obtained by the following theoretical equation:

$${\rm{pH}}={\rm{p}}{{\rm{K}}}_{{\rm{B}}}-\,\log \,(({{\rm{\delta }}}^{11}{{\rm{B}}}_{{\rm{SW}}}-{{\rm{\delta }}}^{11}{{\rm{B}}}_{{\rm{borate}}})/({\rm{\alpha }}\ast {{\rm{\delta }}}^{11}{{\rm{B}}}_{{\rm{borate}}}-{{\rm{\delta }}}^{11}{{\rm{B}}}_{{\rm{SW}}}\ast {10}^{3}\ast ({\rm{\alpha }}-{\rm{1}})))$$

where *p*K_B_ is the dissociation constant of boric acid and is a function of temperature and salinity (and pressure)^68^, δ^11^B_SW_ is the δ^11^B value of seawater, which is constant on glacial–interglacial timescales^59^, and α is the fractionation factor between borate ions and boric acid in the seawater^69,70^.

For the calculation of seawater *p*CO_2_, both pH and one more CO_2_ system parameter need to be known. In this study, the total alkalinity (TA) of the seawater was used for the calculation, and was estimated using a multiple regression equation (a function of temperature and salinity) reported by Lee *et al*.^71^. The *CO2calc* software (version 4.0.9), provided by the U.S. Geological Survey (available at https://www.usgs.gov/software/co2calc), was used to calculate *p*CO_2_ from pH, TA, temperature, and salinity. Dissociation constants for *K*_1_ and *K*_2_ from Lueker *et al*.^72^ and for *K*_B_ from Dickson^68^ were used. Δ*p*CO_2_ (seawater *p*CO_2_ minus atmospheric *p*CO_2_) was calculated from the obtained seawater *p*CO_2_ using contemporary atmospheric *p*CO_2_ reconstructed from Antarctic ice cores^1–3^.

### Estimated calcification depth of planktonic foraminifera

Calcification depths of *G. ruber* and *T. sacculifer* were estimated by comparing theoretically predicted depth profiles of δ^18^O and δ^11^B and measured δ^18^O and δ^11^B values in the Holocene section. For the calculation, depth profiles of annual temperature and salinity from the World Ocean Atlas (data available at https://odv.awi.de/data/ocean/world-ocean-atlas-2009/) were used (Fig. 3a). For CO_2_ system variables, gridded data of dissolved inorganic carbon (DIC) and TA from Global Ocean Data Analysis Project (GLODAP; data available at https://odv.awi.de/data/ocean/glodap-gridded-data/)^43^ were used. Anthropogenic DIC incorporation after the industrial revolution was subtracted from DIC data to estimate pre-industrial DIC, and then all CO_2_ system variables in the pre-industrial era were calculated using the *CO2calc* software (Fig. 3b,c). The theoretical δ^18^O depth profile of calcite was calculated from temperature and δ^18^O_SW_ values obtained from NASA GISS (data available at http://iridl.ldeo.columbia.edu/SOURCES/.NASA/.GISS/.LeGrande_Schmidt2006/.v1p1/). As foraminifera calcite is precipitated in isotopic equilibrium with the ambient seawater, the δ^18^O depth profile of inorganic calcite represents that of foraminifera calcite (Fig. 3d). The δ^11^B depth profile of foraminifera calcite can be predicted from the known temperature, salinity, and pH, as well as an empirical relationship between the δ^11^B of seawater borate ion and that of *G. ruber*^67^ and *T. sacculifer*^13^ (Fig. 3e). As the empirical relationship differs among foraminifera species, depth profiles of the predicted δ^11^B of foraminifera shells differ between *G. ruber* and *T. sacculifer* (Fig. 3e). The estimated calcification depths are 0–75 m (within the mixed layer) and 75–125 m (below the mixed layer; upper thermocline) for *G. ruber* and *T. sacculifer*, respectively.

### Monte Carlo simulation

As many variables are required to calculate pH, *p*CO_2_, and Δ*p*CO_2_, Monte Carlo simulation is useful to calculate the estimation errors of these outputs considering all involved errors. All calculations were performed using the freeware *R* (version 3.0; available at: https://www.r-project.org) with the *seacarb* (v.3.2.8) package for seawater CO_2_ chemistry calculation^73^. The same dissociation constants (*K*_1_, *K*_2_, and *K*_B_) that were used for the seawater CO_2_ chemistry calculation using the CO2calc software were employed. Analytical errors (1σ) of Mg/Ca (3%), δ^18^O (0.1‰), and δ^11^B (0.14‰ and 0.25‰ when ^11^B beam intensity was greater than 0.2 V and less than 0.2 V, respectively) were considered. Estimation errors of atmospheric *p*CO_2_ (2 μatm) reconstructed from Antarctic ice core were also considered. Variables were randomly changed in which errors are normally distributed (mean ± error), and the calculation was repeated 1000 times. Standard deviations of 1000 outputs of pH, *p*CO_2_, and Δ*p*CO_2_ were then calculated, and were regarded as estimation errors (Fig. 2b–d). The error ranged from 0.014 to 0.030 for pH, and from 9 to 32 μatm for both *p*CO_2_ and Δ*p*CO_2_.

## Supplementary information

Supplementary Information

Dataset 1

Dataset 2

Dataset 3
