# Supplementary material for: Equatorial Pacific seawater pCO2 variability since the last glacial period
Source: Sci Rep. 2019 Sep 25;9:13814. doi: 10.1038/s41598-019-49739-0 (PMC6761199; doi:10.1038/s41598-019-49739-0)
Supplement: Supplementary file 1 — Supplementary Information [file 41598_2019_49739_MOESM1_ESM.doc]

**Supplementary Information**

**Equatorial Pacific seawater *p*CO_2_ variability since the last glacial period**

Kaoru Kubota*, Yusuke Yokoyama, Tsuyoshi Ishikawa, Takuya Sagawa, Minoru Ikehara, Toshitsugu Yamazaki

(*) Correspondence to: kaoryu0129@gmail.com

**Potential effects of dissolution on the geochemistry of planktonic foraminifera shells**

Dissolution of the foraminifera shells may influence the initial geochemical information recorded in the shells when the organisms were alive. Dissolution is influenced by multiple factors, such as the saturation state of calcite of bottom water and sedimentation rate.

**Table X1.** Summary of marine sediment cores mentioned in this section.

| **Sediment core** | **Water depth (m)** | **Saturation state of calcite (Ω_calcite_)*** | **Sedimentation rate (cm/kyr)** | **Reference** |
| --- | --- | --- | --- | --- |
| KR05-15 PC01 | 3,226 | 0.87 | 4.5 | This study |
| MD97-2138 | 1,960 | 1.20 | 11.0 | ref. 1 |
| ERDC-92 | 1,598 | 1.31 | 1.6 | ref. 2 |
| ODP806B | 2,520 | 1.03 | 2.9 | ref. 3 |

(*) If Ω_calcite_ is less than 1, inorganically precipitated calcite starts to dissolve.

For *G. ruber*, Dekens *et al*.^4^ and Fehrenbacher *et al*.^5^ reported that the δ^18^O of the shell is insensitive to dissolution. This is supported by the fact that the δ^18^O variation of core KR05-15 PC01 since the last glacial period shows good agreement with that reported by de Garidel-Thoron *et al*.^1^ based on a nearby sediment core, MD97-2138, from the equatorial Pacific, which is characterized by excellent preservation of foraminifera shells due to a high sedimentation rate (11.0 cm/ky) (Fig. S5). Foster^3^ and Ni *et al*.^6^ report no significant effect of dissolution on δ^11^B: Foster^3^ measured the δ^11^B of *G. ruber* collected from marine sediment core tops from sites in the Pacific and Atlantic (including the Ontong Java Plateau site, ODP806B, Fig. 1), and found no discernible difference among different water depths (Ω_calcite_ of bottom water ranges 1.0–1.5); Ni *et al.*^6^ measured the δ^11^B of different shell size fractions (200–250, 250–300, 300–355, and 355–425 μm) of *G. ruber* collected from core-top sedimentary material from the Ontong Java Plateau (Fig. 1), which is characterized by a low sedimentation rate (2.9 cm/ky), and found no difference between them.

However, for *T. sacculifer*, a significant effect of dissolution on both δ^18^O and δ^11^B has been reported^6,7^. This is most likely due to the presence of the sac-like final chamber and the precipitation of a gametogenic calcite coating during the last life stage of this animal^6-12^, as well as its selective preservation^7^. The sac-like final chamber and other gametogenic calcite coating of the shell are thought to precipitate at thermocline depths based on careful observation of cultured and fossil samples. During the reproduction, they migrate into the thermocline depth and release gametes, which is governed by lunar cycles^9,11,12^. Due to the higher resistance of the sac-like final chamber to dissolution after deposition in the marine sediment, the effect of dissolution becomes larger as it progresses^6,7^. Consequently, as *T. sacculifer* shells dissolve, the recorded environmental signal becomes more "thermocline-like": δ^18^O and δ^11^B values become isotopically heavier (suggesting lower water temperature) and lighter (suggesting lower pH), respectively. Nevertheless, it is noteworthy that δ^18^O variation, and thus the estimation of mean calcification depth, of *T. sacculifer*, are consistent between de Garidel-Thoron *et al*.^1^ and the present study, even though de Garidel-Thoron *et al*.^1^ used "without sac" *T. sacculifer* individuals, which are less susceptible to the effect of dissolution (Table X1, Fig. S6). This suggests that the *T. sacculifer* with a sac-like final chamber used in this study records the same ocean conditions as *T. sacculifer* without a sac-like final chamber, which is likely due to the modest sedimentation rate of KR05-15 PC01 (4.5 cm/ky), despite the fact that Ω_calcite_ of bottom water is less than 1 (0.87). Similarly, Foster^3^ found no discernible difference in the δ^11^B values of *T. sacculifer*, as also reported for *G. ruber*, at least when Ω_calcite_ of bottom water is greater than 1. As δ^11^B values of *T. sacculifer* from core KR05-15 PC01 are consistent with those of *T. sacculifer* from core-top material (mean age is centered on ~8 ka) from the Ontong Java Plateau site (ODP806B, Ω_calcite_ = 1.03) reported by Foster^3^ (Fig. S7), it follows that, like δ^18^O, the δ^11^B of *T. sacculifer* used in this study is not significantly affected by dissolution. It is also noteworthy that large *T. sacculifer* individuals are less influenced by dissolution, because of a relative proportion of sac versus other chambers are smaller in large individuals^7^.

From the above discussion, it is safe to conclude that both the δ^18^O (and thus the estimation of mean calcification depth) and δ^11^B values of *G. ruber* and *T. sacculifer* are free from dissolution effects. However, we need to consider the effect of water temperature reconstruction on *p*CO_2_ estimation, because several Mg/Ca–T equations have been proposed, and temperature is one of the most important factors affecting seawater *p*CO_2_ reconstruction. We used the equation proposed by Sagawa *et al*.^13^; however, a different choice of Mg/Ca–T equation may lead to the calculation of a different *p*CO_2_ value. Thus, we additionally used two other equations, in order to test whether the calculation of seawater *p*CO_2_ was affected by the choice of Mg/Ca–T equation^4,13,14^ (Fig. S8). The maximum difference in *p*CO_2_ was 24 μatm, which is within the estimation error of *p*CO_2_ (9–32 μatm, 1σ). Therefore, our conclusion is unaffected by the choice of Mg/Ca–T equation. It is noteworthy that among the three equations, that of Dekens *et al*.^4^ includes a water depth correction term, which means that dissolution is considered in this equation. It follows that effect of dissolution on Mg/Ca is negligible, at least for the *p*CO_2_ reconstruction in this study.

In addition, we investigated a potential influence of surface seawater pH changes on Mg/Ca-thermometer, which is suggested by previous studies (e.g., refs. 15–17), and its influence on *p*CO_2_ estimation. This is because since the last glacial period surface pH likely decreased due to atmospheric *p*CO_2_ increases, which subsequently influences temperature reconstruction based on Mg/Ca proxy, and thus pH and *p*CO_2_ reconstructions. We employed a recently released source-code for R software by Gray and Evans^18^ which considers the pH-effect on Mg/Ca-thermometer and enables an iteration of temperature and pH calculation from Mg/Ca and δ^11^B until a stable solution emerges. In the calculation we chose the Mg/Ca-T equation of *G. ruber* (white) recently compiled by Gray *et al.*^19^, "grbw_gray2018". As Mg/Ca record of KR05-15 PC01 shows lower value than that of MD97-2137, especially during the Holocene interval (Fig. S5a), we corrected the original Mg/Ca values by +20%, which may be due to a partial dissolution of *G. ruber* shell. Without this correction, the temperature reconstruction became ~2°C lower constantly through the record. From temperature and pH, we further calculated *p*CO_2_ using CO2sys software assuming constant total alkalinity, as its influence is minor (see the following "How total alkalinity estimation influences the calculation of *p*CO_2_? " for the detailed discussion). The results showed that a relatively large difference during the Holocene interval: namely, temperature and pH difference of ~1°C and ~0.02 pH unit, respectively (Fig. S9a,b). During the other interval, on the other hand, both temperature and pH reconstruction showed no significant difference (Fig. S9c,d). It is noteworthy that the calculated *p*CO_2_ showed no significant difference: namely only 12 μatm in maximum, which is with in estimation error of *p*CO_2_ (9–32 μatm, 1σ). Thus, we concluded that the consideration of pH effect on Mg/Ca-thermometer on *p*CO_2_ reconstruction is negligible, at least for this study site.

**Comparison of *T. sacculifer* δ^11^B records between core KR05-15 PC01 and core ERDC-92**

Here, we discuss the discrepancy between our new *T. sacculifer* δ^11^B records with those previously reported by Palmer and Pearson^2^ in the WEP (Fig. S7). The absolute δ^11^B values of *T. sacculifer* obtained in this study are in line with those determined by Foster^3^ for the core-top sediment in the Ontong Java Plateau (Fig. S7a). In contrast, the values reported by Palmer and Pearson^2^ are much higher, by as much as approximately 6‰. This is highly likely due to the different analytical methodologies used to determine isotopic composition: Foster^3^ used MC-ICPMS, and Palmer and Pearson^2^ used negative thermal ionization mass spectrometry (N-TIMS). δ^11^B values determined by the N-TIMS method are often higher than those measured by MC-ICPMS (e.g., refs. 20-22).

If we focus on trend of variability, rather than absolute δ^11^B values, records of both this study and Palmer and Pearson^2^ show the common feature that δ^11^B values during the last glacial period are higher by about 1‰ than those during the Holocene (Fig. S7a,b). However, δ^11^B variability during the last deglaciation is quite different: local minima and local maxima appear at totally different timings. For instance, ERDC-92 shows local minimum centered on ~15 ka (based on which Palmer and Pearson^2^ argued that the WEP was a CO_2_ source during the last deglaciation), but KR05-15 PC01 does not show such a minimum (Fig. S7a,b). It is unlikely that this is a result of age model uncertainties in the KR05-15 PC01 and ERDC-92 cores, because a sufficient number of ^14^C dates were obtained for these cores (Fig. S7a,b). In addition, we found no significant changes in sedimentation rate of both cores through the record (Fig. S6c). Another explanation is the different locations of these cores: as there is ~1500 km between the KR05-15 PC01 and ERDC-92 coring sites, there is a possibility that deglacial oceanographic conditions differed between the two sites. As the ERDC-92 site is closer to the equatorial Pacific upwelling region, depth profiles of pH and *p*CO_2_ show respectively lower and higher values compared with those in KR05-15 PC01 sites (Fig. S7c,d). However, the predicted δ^11^B values for *T. sacculifer* in the pre-industrial period differ by only 0.4‰. Thus, it is more likely that the δ^11^B records of ERDC-92 were altered by dissolution due to a very low sedimentation rate (1.6 cm/ky: Table X1, Fig. S6). Based on analysis of core-top materials of Ontong Java Plateau sites with low sedimentation rates, Honisch and Hemming^7^ and Ni *et al*.^6^ suggested that dissolution of the shells alters the original δ^11^B values, especially when *T. sacculifer* with a sac-like final chamber is used. This notion is supported by the fact that the δ^18^O values of *T. sacculifer* in core ERDC-92 are approximately 0.5‰ higher throughout the record than those of cores KR05-15 PC01 and MD97-2138 (Fig. S6). This trend is consistent with the finding of Dekens *et al*.^4^ that δ^18^O values of *T. sacculifer* shells become isotopically heavier as they dissolve.

**How total alkalinity estimation influences the calculation of *p*CO_2_?**

There are mainly two ways to estimate TA changes on glacial-interglacial timescales. One proposes an extrapolation of a relationship between salinity/temperature/TA seen in the present surface ocean to the past (e.g., refs. 2,20,23,24). A linear relationship between salinity and δ^18^O_SW_ is also assumed in this approach. The other proposes modest TA changes on glacial-interglacial timescales which is based on the earth system model output, because there is no direct way to reconstruct secular changes of TA (e.g., refs. 25-27). This approach is skeptical to the assumption that a relationship between salinity/temperature/TA is held constant through time. To see the difference, we made a calculation according to these two approaches (Fig. S10 & S11): (1) TA is calculated from regional temperature and salinity reconstruction given that the regression is held constant through time (that is the method described in “Calculation of pH and *p*CO_2_”); (2) TA takes a range in modern-day alkalinity at the site plus an estimate of the glacial alkalinity increase (+75 μmol/kg), and modern-day alkalinity at the site minus 25 μmol/kg, and salinity changes in accordance with sea level changes due to ice sheet volume changes.

A distinct difference between these two scenarios is that salinity values during the LGM: The former show a lower salinity and relatively wet condition during the LGM, and the latter reconstruct a higher salinity during the LGM because of a large fresh water (that is ice sheet) on land. The former approach considers a possibility of regional changes of precipitaion/evaporation balance (thus S, TA, and δ^18^O_SW_ changes) in the equatorial Pacific region on glacial-interglacial timescales. The former approach seems more realistic in the equatorial region, because significant hydrological changes occurred on glacial-interglacial timescales due to a fluctuation of inter-tropical convergent zone (ITCZ) (e.g., refs. 28-30). The reconstructed δ^18^O_SW_ changes based on the former approach are consistent with those of the previous study for both *G. ruber* and *T. sacculifer*^1^ (Fig. S9b & S10b). However, we found that, no matter what the TA estimation is, *p*CO_2_ reconstruction does not differ significantly (Fig. S9d & Fig. S10d) and our conclusion is unaffected.


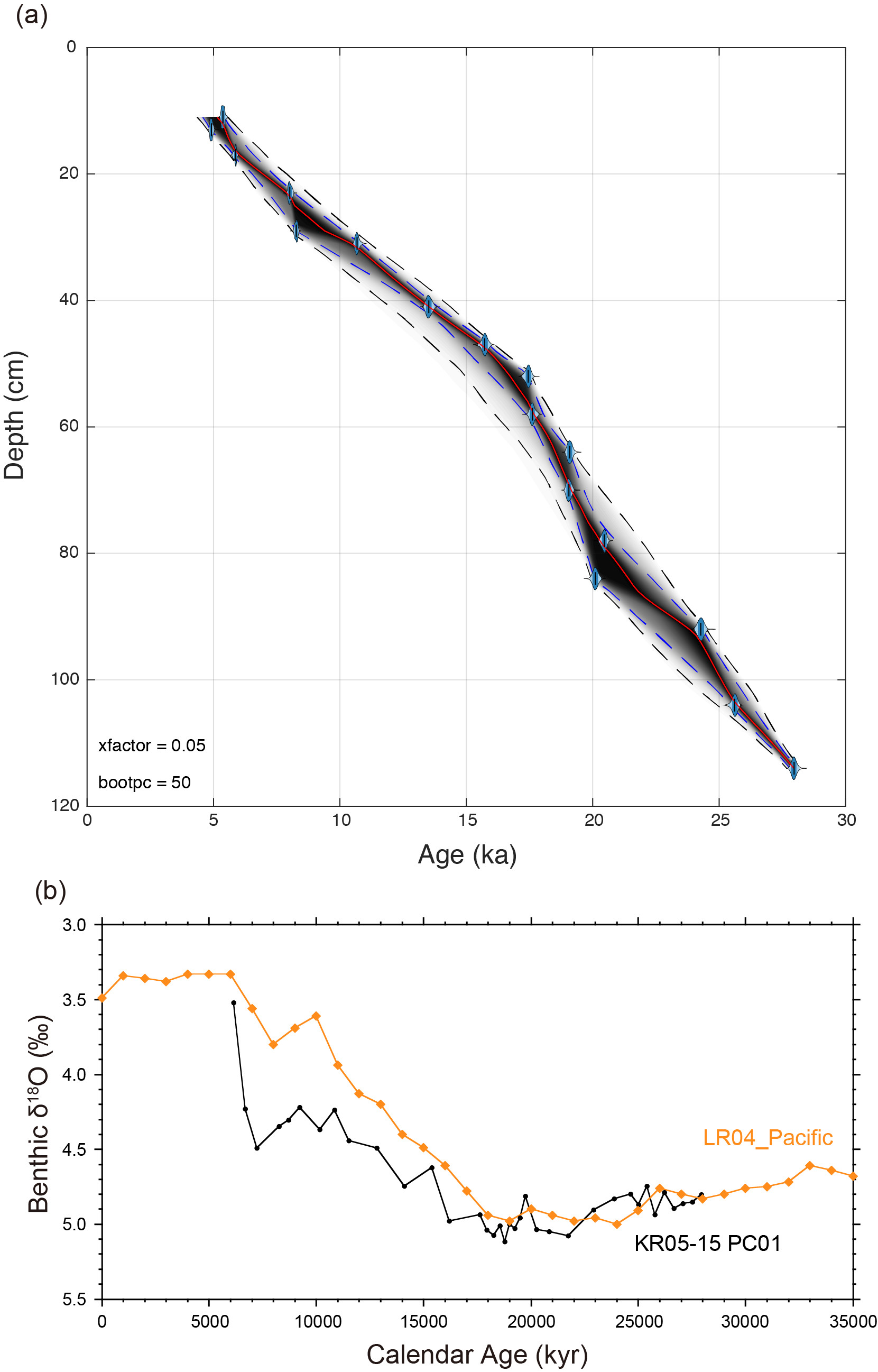
**Supplementary Fig. S1.**

(**a**) Age-depth model of core KR05-15 PC01. Blue probability density functions indicate the radiocarbon age-depth constraints. The grey cloud indicates the probability density cloud of the age-depth model, whereby darker colors indicate higher age-depth probability. The red line indicates the age-depth model median. The dashed black and blue lines indicate the age-depth model 2σ and 1σ confidence intervals, respectively. (**b**) Comparison of δ^18^O values of benthic foraminifera, *Uvigerina* spp., between KR05-15 PC01 (black) and a stack of Pacific sediment cores (yellow)^31^.


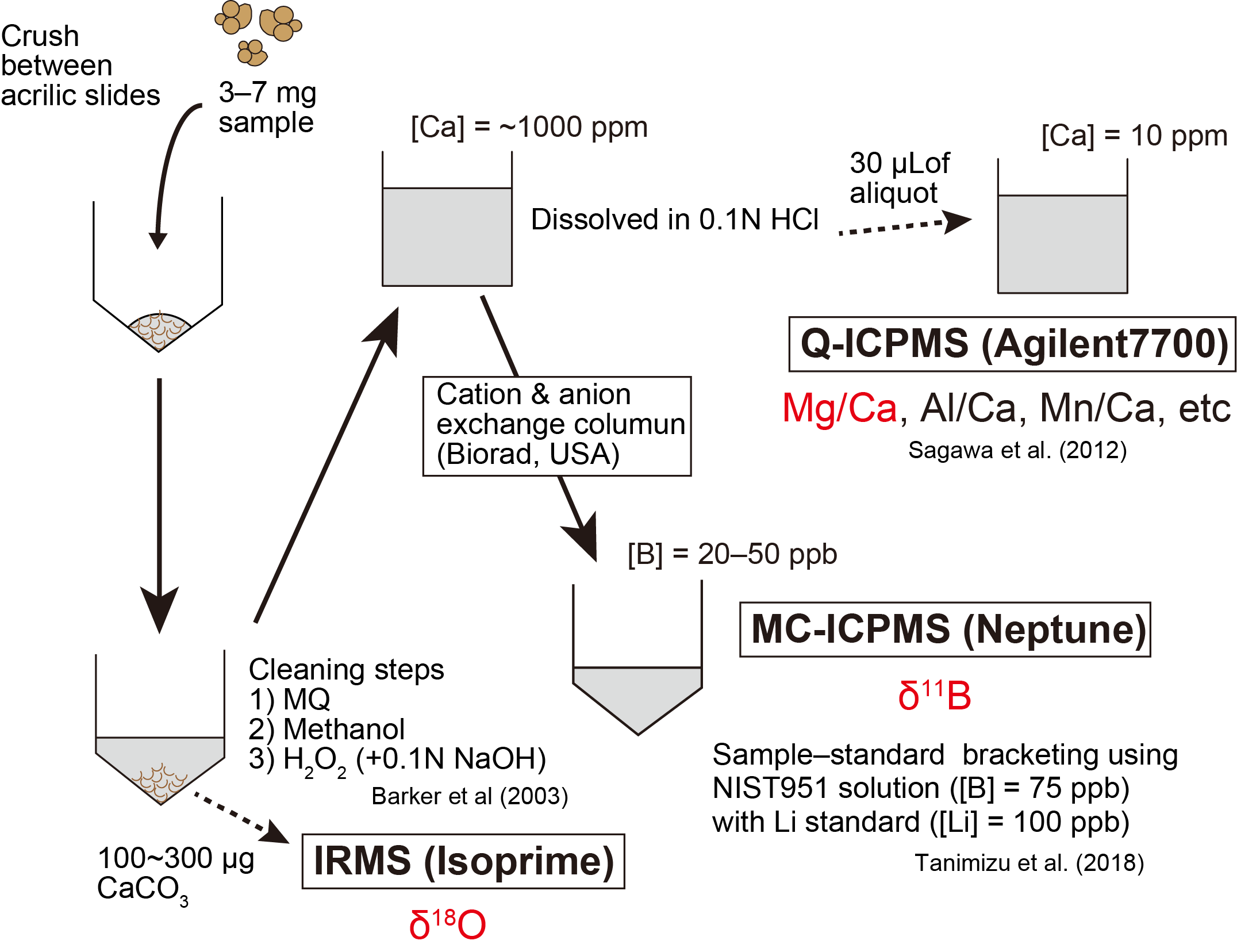


Supplementary Fig. S2.

Schematics of the experimental procedures for δ^18^O, Mg/Ca, and δ^11^B analysis from one sample.


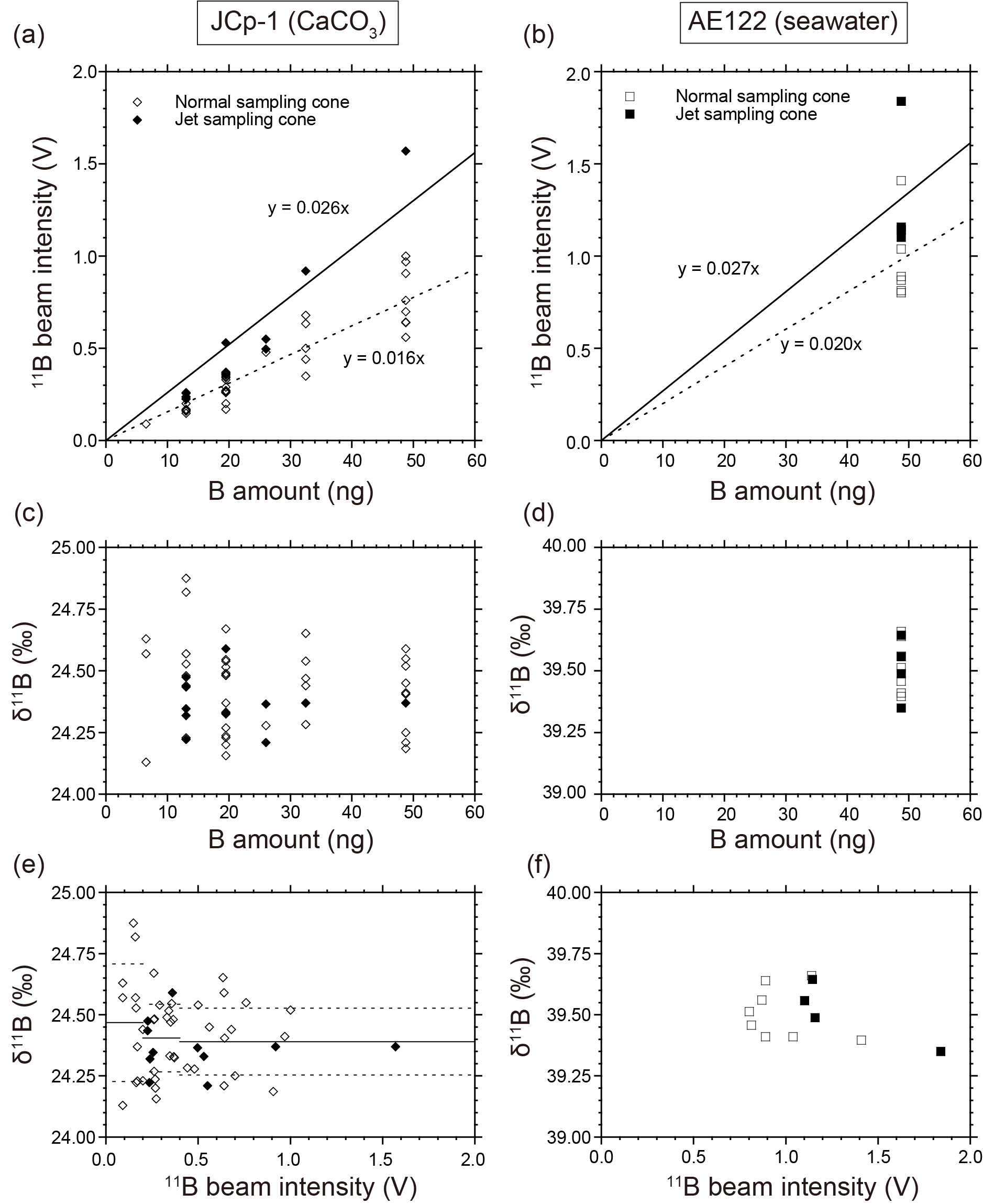


Supplementary Fig. S3.

(**a**) Relationship between ^11^B beam intensity of the samples and the amount of boron in JCp-1. ^11^B beam intensity increased when employing a Jet sampling cone in place of a normal sampling cone. (**b**) Same as part (a), but for AE122. (**c**) Measured δ^11^B values of JCp-1 versus amount of boron. (**d**) Same as (c), but for AE122. (**e**) Measured δ^11^B values of JCp-1 versus ^11^B beam intensity. The solid line indicates the average value, and dashed lines indicates the ±1σ range. (**f**) Same as (e), but for AE122.


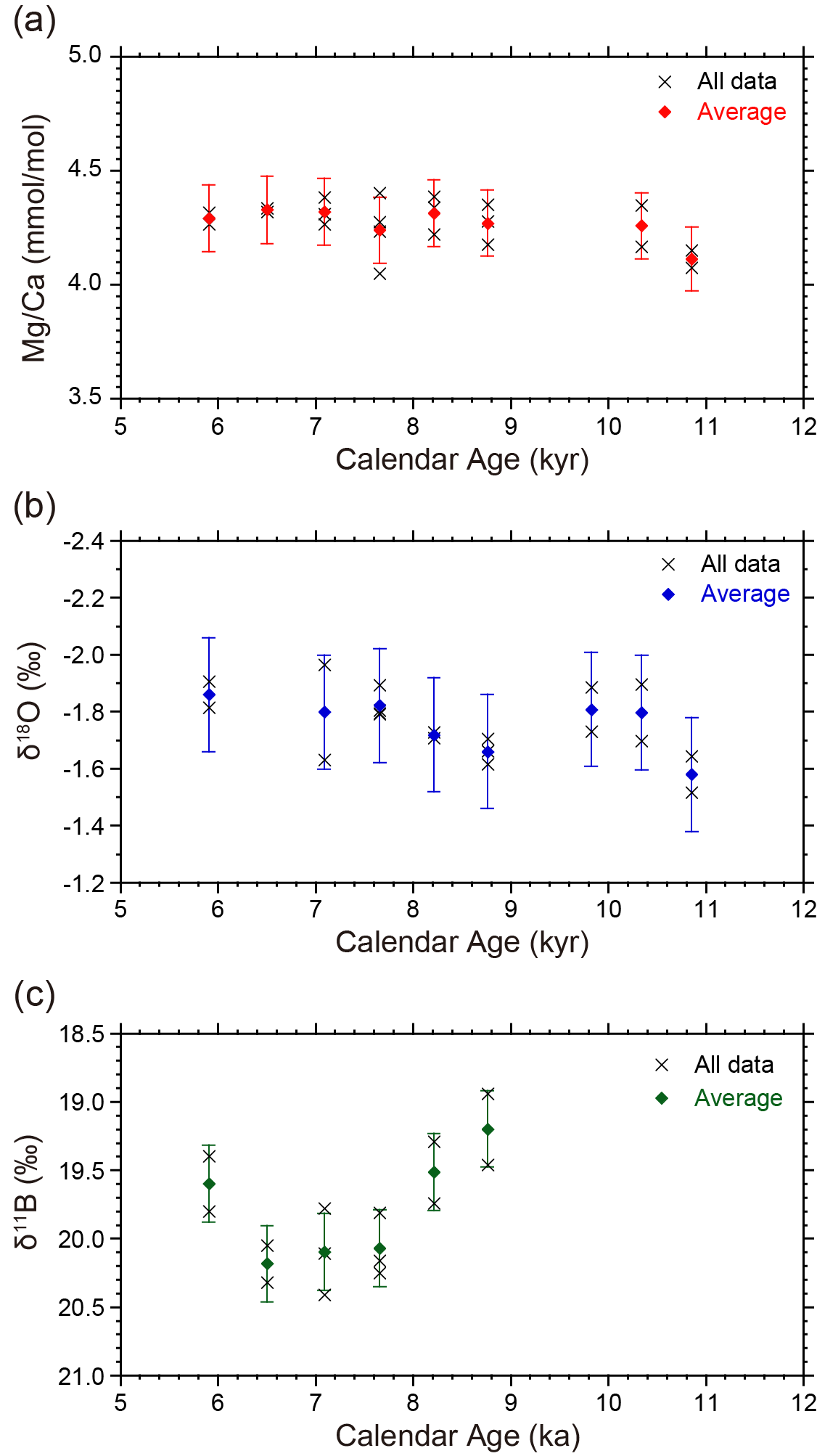


Supplementary Fig. S4.

Replicate analysis of (**a**) Mg/Ca, (**b**) δ^18^O, and (**c**) δ^11^B of *T. sacculifer* in core KR05-15 PC01. The Holocene section was used for the analysis, as foraminifera shells were abundant during this period. Each data point is plotted as a cross, and averages are plotted as diamonds with analytical errors (2σ).


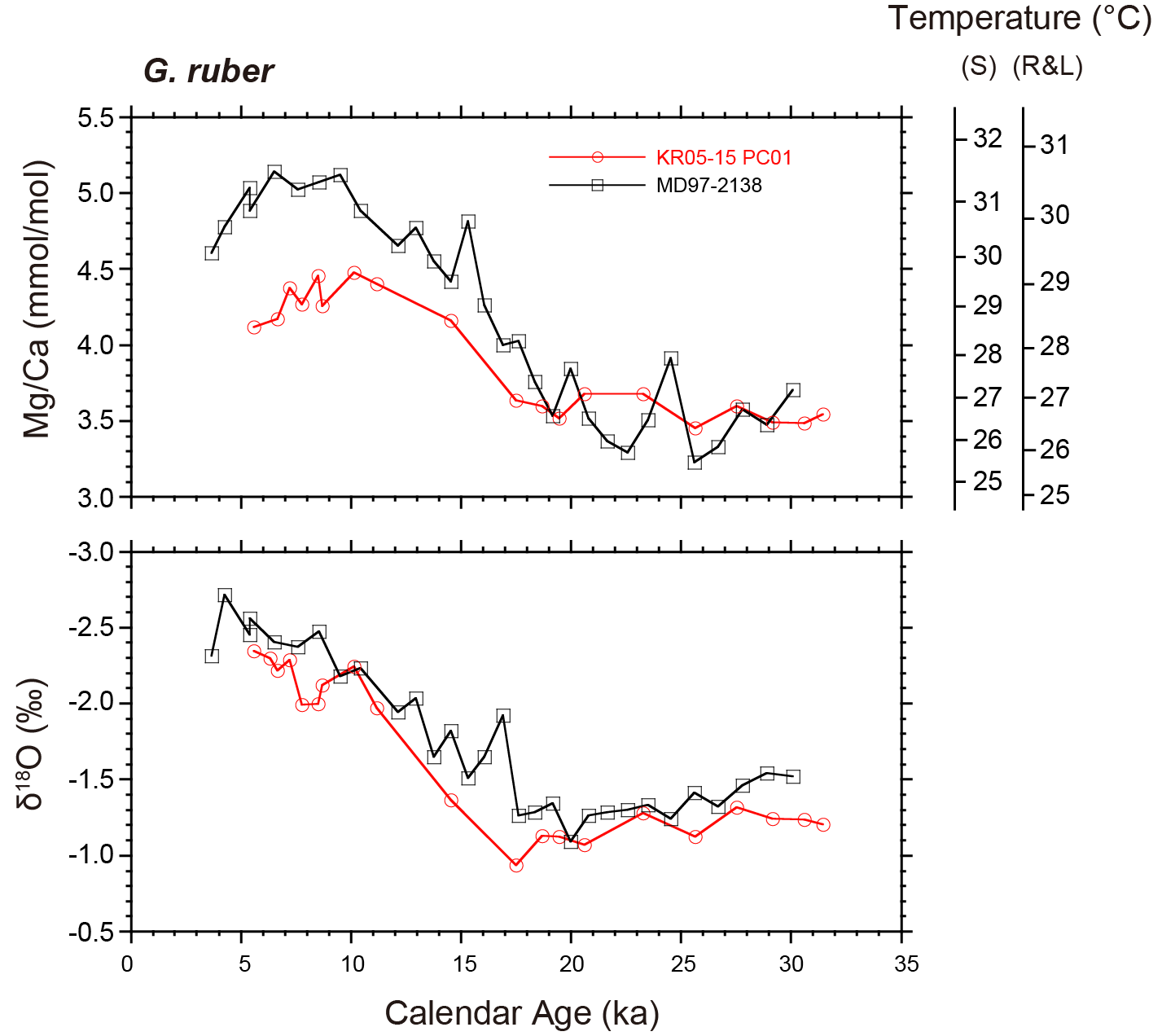


Supplementary Fig. S5.

(**a**) Mg/Ca and (**b**) δ^18^O records of *G. ruber* from WEP sediment cores. Temperature reconstruction using different Mg/Ca-T equations are indicated in **a**. (S) denotes Sagawa *et al*.^13^, and (R&L) denotes Rosenthal and Lohman^14^.


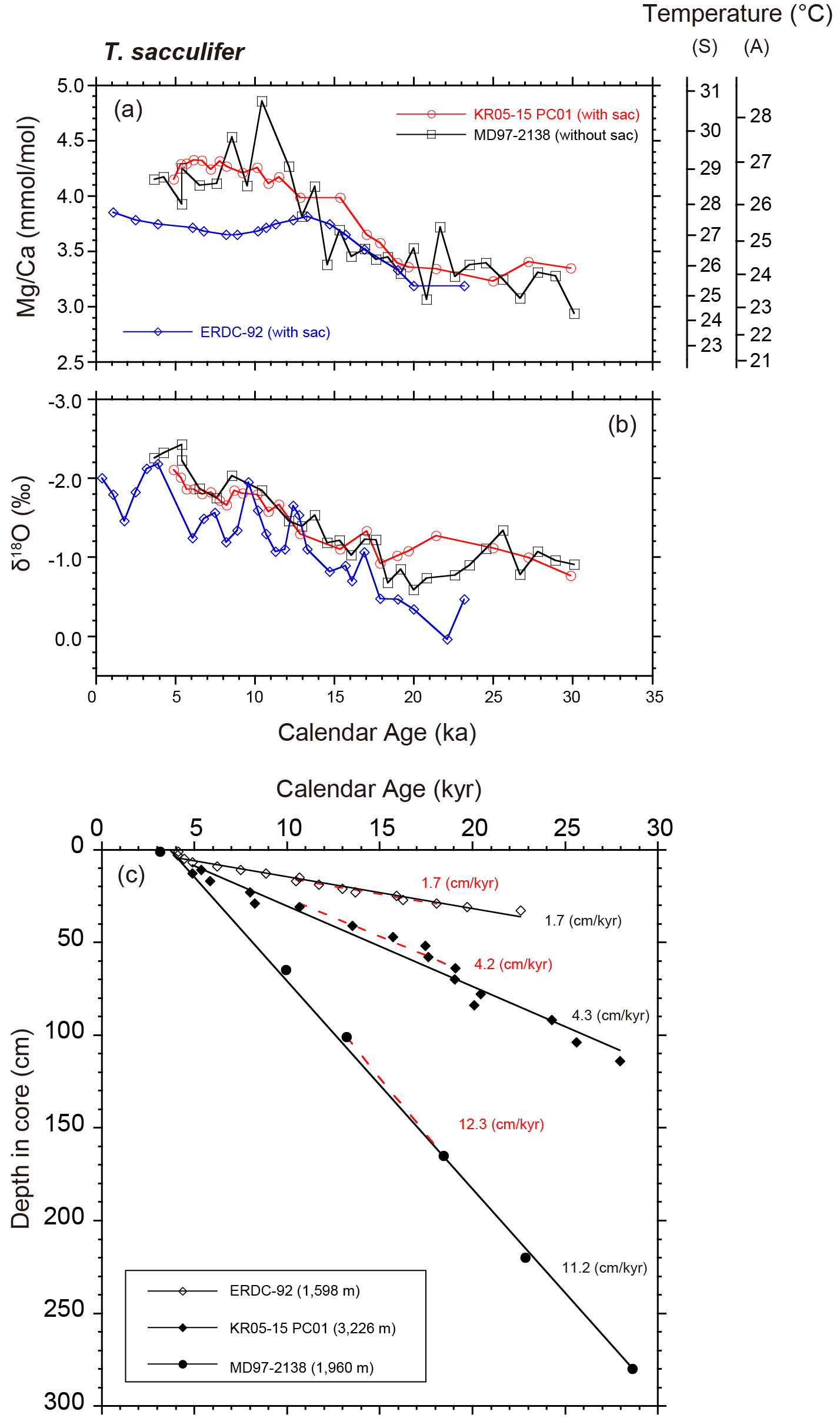
Supplementary Fig. S6.

(**a**) Mg/Ca and (**b**) δ^18^O records of *T. sacculifer* from WEP sediment cores. Temperature reconstruction using different Mg/Ca-T equations are indicated in **a**. (S) denotes Sagawa *et al*.^13^, and (A) denotes Anand *et al*.^32^. Note that *T. sacculifer* shells with a sac-like final chamber were analyzed in KR05-15 PC01 and ERDC-92 (ref. 2), and those without a sac-like final chamber were analyzed in MD97-2138 (ref. 1). (**c**) Age-depth relationship of three sediment cores with linear regression lines (black). A regression lines during the period corresponding to the last deglaciation is indicated in red dashed lines.


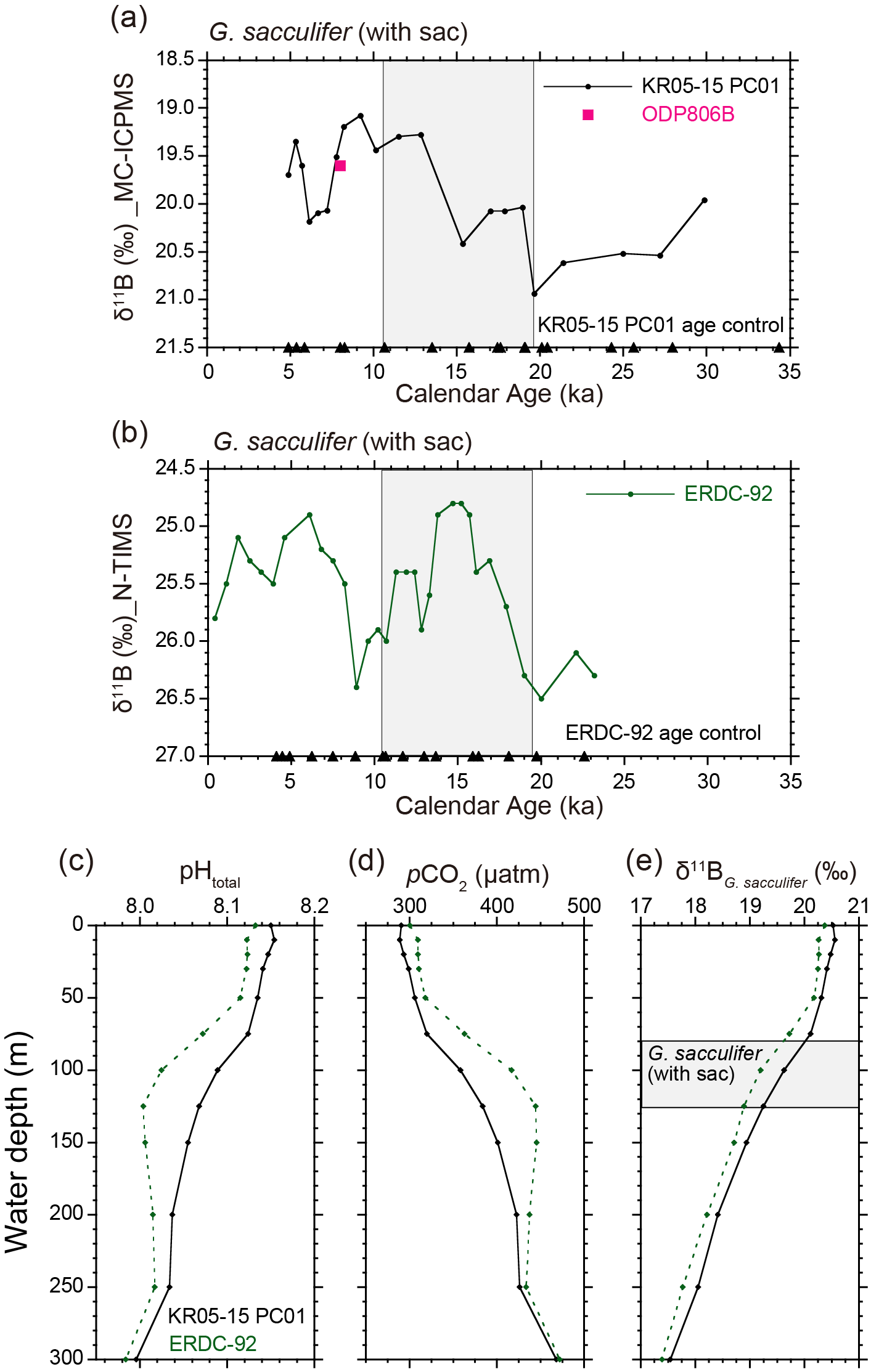
Supplementary Fig. S7.

δ^11^B values of *T. sacculifer* from (**a**) core KR05-15 PC01 and (**b**) core ERDC-92 (ref. 2). In (**a**), the δ^11^B values of *T. sacculifer* with a sac-like final chamber collected from core-top material of Ontong Java Plateau sediment (ODP806B) reported by Foster^3^ are also plotted. Radiocarbon-based age controls are indicated by triangles. The shaded areas indicate the period corresponding to the last deglaciation. Also shown are pre-industrial depth profiles of (**c**) pH and (**d**) *p*CO_2_, of seawater, and (**e**) expected δ^11^B values of *T. sacculifer* shells at the coring locations of KR05-15 PC01 (solid black line) and ERDC-92 (dashed green line). The shaded area in (e) indicates the estimated calcification depth in the WEP (from Fig. 3).


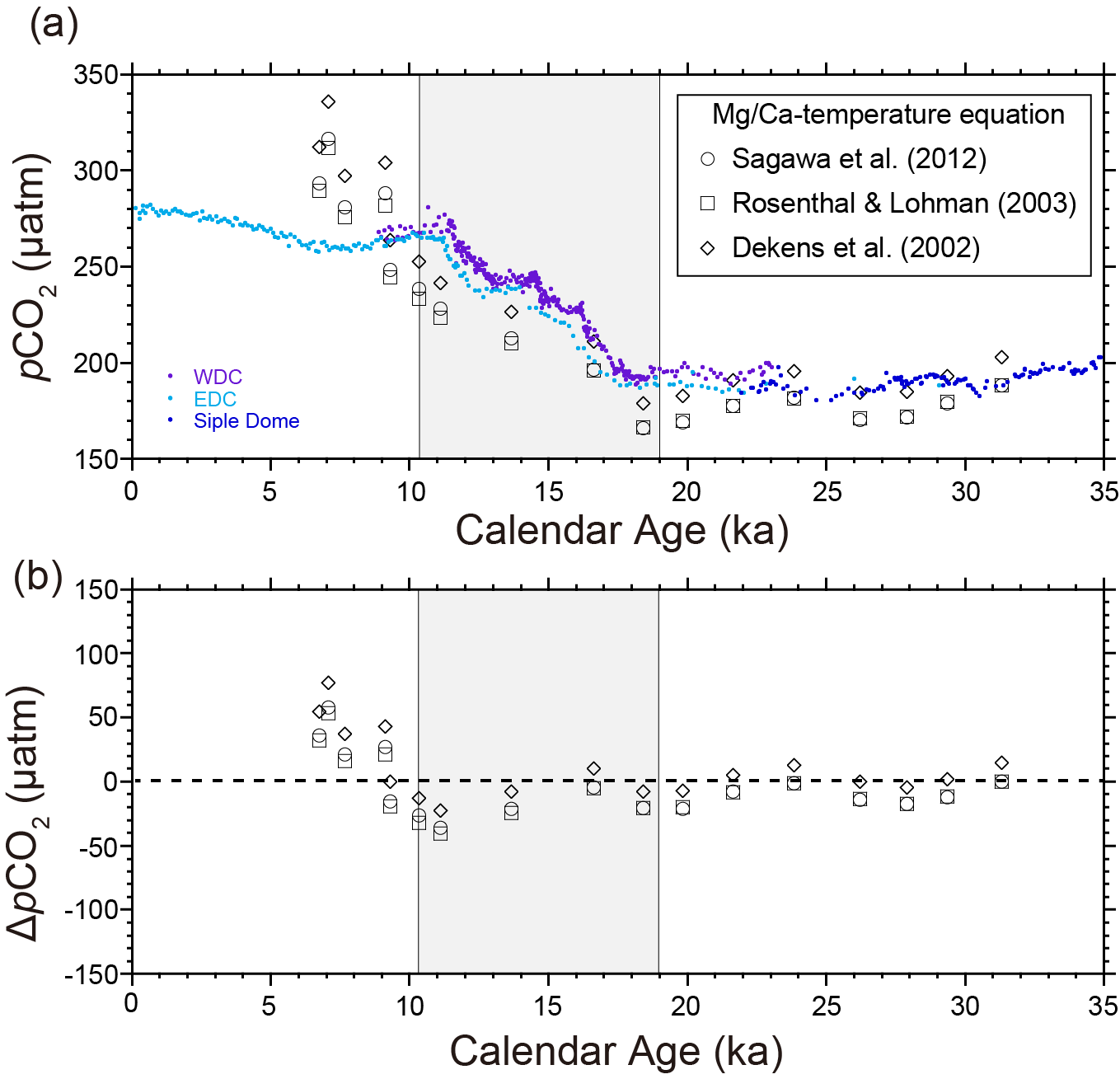
Supplementary Fig. S8.

(**a**) Seawater *p*CO_2_ reconstructions using different Mg/Ca–temperature equations. Also plotted is atmospheric *p*CO_2_ reconstructed from Antarctic ice cores (West Antarctic Ice Sheet Divide ice core, WDC; Siple Dome; EPICA Dome C, EDC)^33-35^. The shaded area indicates the period of the last deglaciation. (**b**) Same as (a), but for Δ*p*CO_2_ reconstructions.


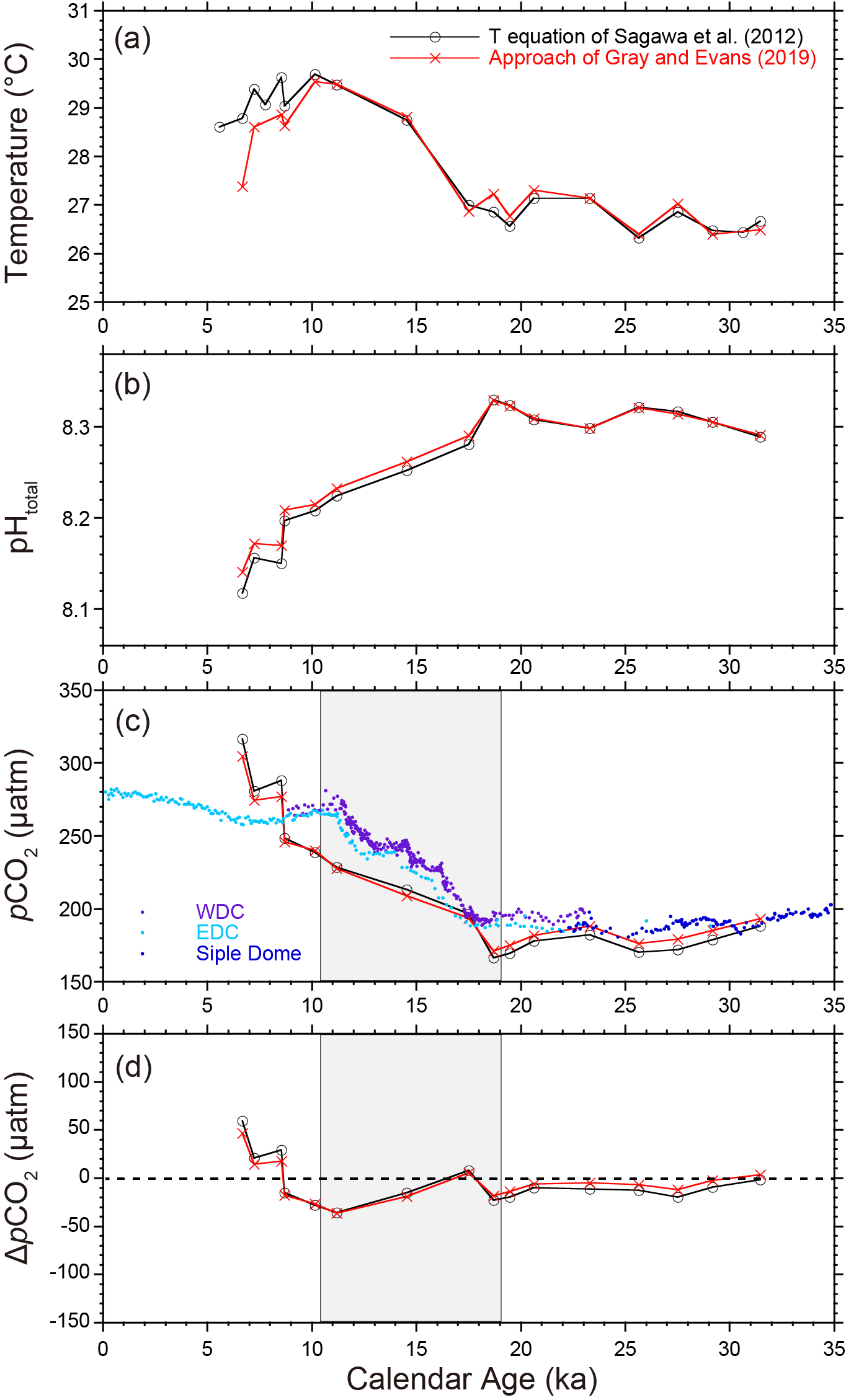
Supplementary Fig. S9.

(**a**) Temperature reconstruction using Mg/Ca-T equation reported by Sagawa *et al.*^13^ (black circles) and an approach proposed by Gray and Evans^18^ in which pH effects on Mg/Ca-T relationship is considered (red crosses). (**b,c,d**) Same as in (a**)**, but for pH, *p*CO_2_, Δ*p*CO_2_ reconstructions, respectively. Also plotted in **c** is atmospheric *p*CO_2_ reconstructed from Antarctic ice cores (West Antarctic Ice Sheet Divide ice core, WDC; Siple Dome; EPICA Dome C, EDC)^33-35^. The shaded area in (c) and (d) indicates the period of the last deglaciation.


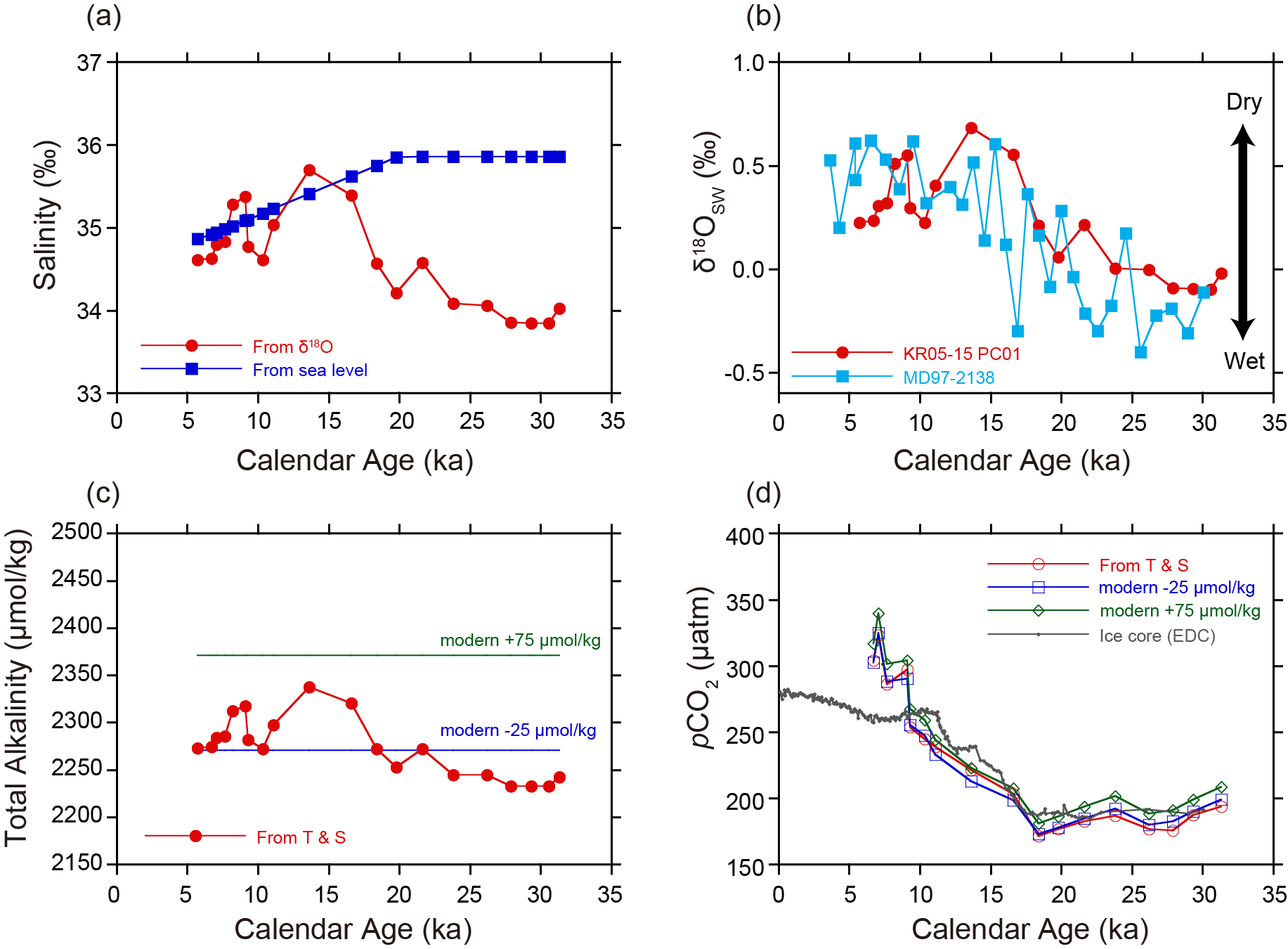


Supplementary Fig. S10.

(**a**) Salinity estimation based on different scenarios: Red, estimation from Mg/Ca and δ^18^O values of *G. ruber* shell; blue, estimation from sea level changes in which 135 m sea level changes are equivalent to 1 psu salinity increase. (**b**) Reconstruction of δ^18^O_SW_ from a combination of Mg/Ca and δ^18^O values of *G. ruber* collected from two marine sediment cores. (**c**) Different TA scenarios: red, TA reconstructed from an extrapolation of modern regression in which TA is a function of temperature and salinity^18^; green and blue, modern TA plus 75 and minus 25, respectively. (**d**) *p*CO_2_ reconstruction based on three different TA estimation scenarios (legends are same as in **c**). EDC Ice core *p*CO_2_ reconstruction is also plotted as gray line with dots^35^. Note that different TA estimation lead to non-significant difference in *p*CO_2_ estimation.


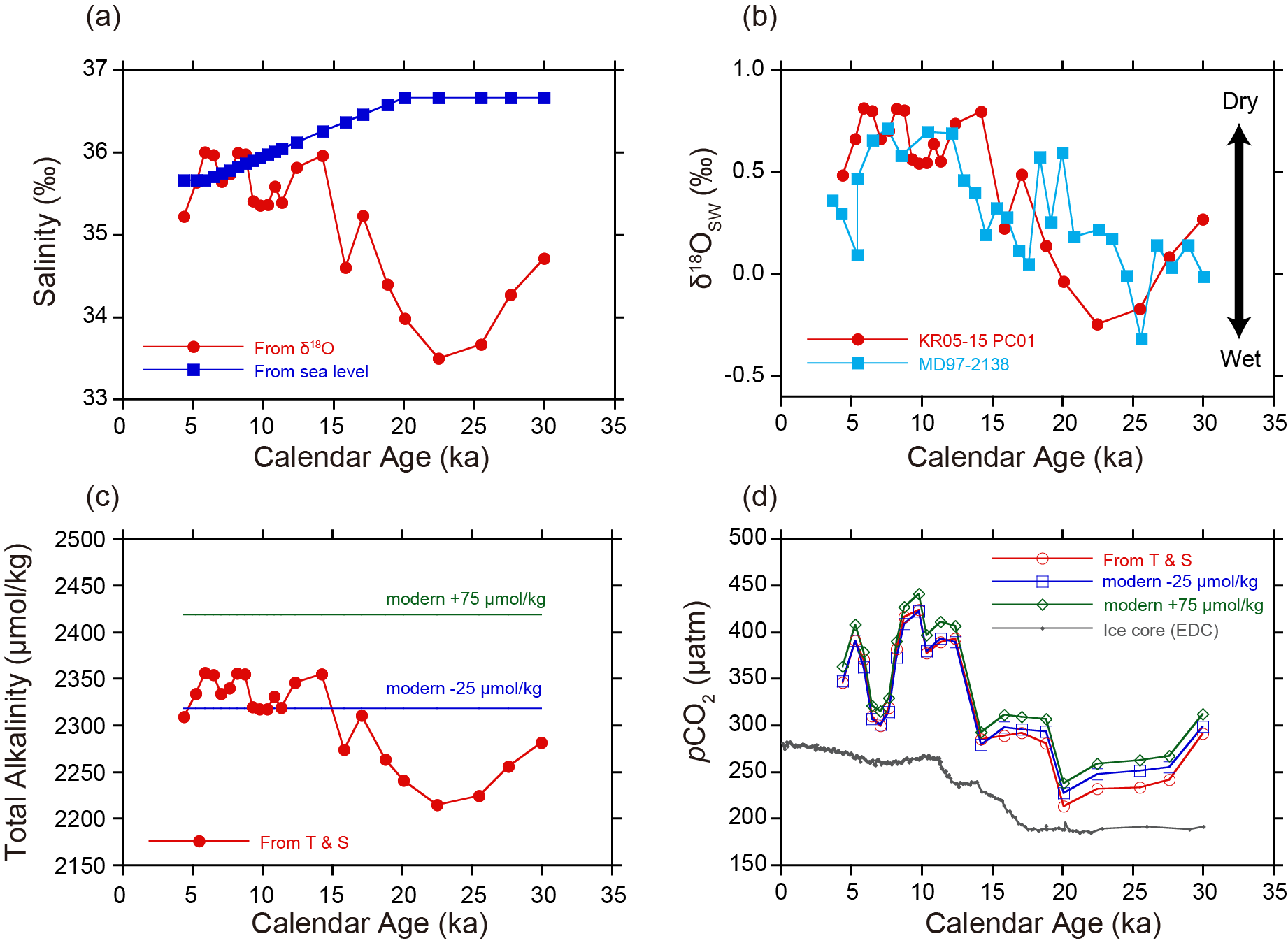


Supplementary Fig. S11.

Same as in Fig. S10, but for *T. sacculifer*.

Supplementary Table 1.

Radiocarbon dates.

Supplementary Table 2.

δ^18^O values of *Uvigerina* spp.

Supplementary Table 3.

δ^11^B values of *G. ruber* and *T. sacculifer*, as well as reconstructed temperature, δ^18^O_SW_, salinity, TA, pH, seawater *p*CO_2_, and Δ*p*CO_2_.

**References**

1. de Garidel-Thoron, T. *et al.* A multiproxy assessment of the western equatorial Pacific hydrography during the last 30 kyr. *Paleoceanography* 22, PA3204, doi:10.1029/2006PA001269 (2007).
2. Palmer, M. R. & Pearson, P. N. A 23,000-Year Record of Surface Water pH and *p*CO_2_ in the Western Equatorial Pacific Ocean. *Science* 300, 480–482 (2003).
3. Foster, G. L. Seawater pH, pCO_2_ and [CO_3_^2-^ ] variations in the Caribbean Sea over the last 130 kyr: A boron isotope and B/Ca study of planktic foraminifera. *Earth Planet. Sci. Let.* 271, 254–266 (2008).
4. Dekens, P. S., Lea, D. W., Pak, D. K. & Spero, H. J. Core top calibration of Mg/Ca in tropical foraminifera: Refining paleotemperature estimation. *Geochem. Geophys. Geosys*. 3, 10.1029/2001GC000200 (2002).
5. Fehrenbacher, J. & Martin, P. Western equatorial Pacific deep water carbonate chemistry during the Last Glacial Maximum and deglaciation: Using planktic foraminiferal Mg/Ca to reconstruct sea surface temperature and seafloor dissolution. *Paleoceanography* 26, PA2225, doi:10.1029/2010PA002035 (2011).
6. Ni, Y. *et al.* A core top assessment of proxies for the ocean carbonate system in surface-dwelling foraminifers. *Paleoceanography* 22, http://dx.doi.o rg/10.1029/2006PA001337 (2007).
7. Hönisch, B., Hemming, N. G. Ground-truthing the boron isotope-paleo-pH proxy in planktonic foraminifera shells: Partial dissolution and shell size effects. Paleoceanography 19, PA4010, doi:10.1029/2004PA001026 (2004).
8. Rippert, N. *et al*. Constraining foraminiferal calcification depths in the western Pacific warm pool. *Mar. Micropaleontol.* 128, 14-27 (2016).
9. Rebotim, A. *et al.* Factors controlling the depth habitat of planktonic foraminifera in the subtropical eastern North Atlantic. *Biogeosciences* 14, 827–859 (2017).
10. Be, A. W. H. Gametogeniccalcification in a spinose planktonic foraminifera, Globigerinoides sacculifer (Brady), Marine Micropaleontology 5, 283–310 (1980).
11. Bijma, J. & Hemleben, C. Population dynamics of the planktic foraminifer *Globigerinoides sacculifer* (Brady) from the central Red Sea. *Deep-Sea Res., Part 1, Oceanogr. Res. Pap.* 41, 485–510 (1994).
12. Erez, J., Almogi-Labin, A. & Avraham, S. On the life history of planktonic foraminifera: lunar reproduction cycle in *Globigerinoides sacculifer* (Brady), *Paleoceanogr*aphy 6 295–306 (1991).
13. Sagawa, T., Yokoyama, Y., Ikehara, M. & Kuwae, M. Shoaling of the western equatorial Pacific thermocline during the last glacial maximum inferred from multispecies temperature reconstruction of planktonic foraminifera. *Palaeogeogr. Palaeoclim. Palaeoecol.* 346–347, 120–129 (2012).
14. Rosenthal, Y., Lohmann, G. P. Accurate estimation of sea surface temperatures using dissolution-corrected calibrations for Mg/Ca paleothermometry, *Paleoceanography* 17, 1044, doi:10.1029/2001PA000749 (2002).
15. Lea, D. W., Mashiotta, T. A. & Spero, H. J. Controls on magnesium and strontium uptake in planktonic foraminifera determined by live culturing. *Geochim. Cosmochim. Acta* 63, 2369–2379 (1999).
16. Russell, A. D., Hönisch, B., Spero, H. J. & Lea, D. W. Effects of seawater carbonate ion concentration and temperature on shell U, Mg, and Sr in cultured planktonic foraminifera. *Geochim. Cosmochim. Acta* 68, 4347–4361 (2004).
17. Allen, K. A. *et al.* Trace element proxies for surface ocean conditions: A synthesis of culture calibrations with planktic foraminifera. *Geochim. Cosmochim. Acta* 193, 197–221 (2016).
18. Gray, W. R. & Evans, D. Nonthermal influences on Mg/Ca in planktonic foraminifera: A review of culture studies and application to the last glacial maximum. *Paleoceanogr. Paleoclimatol.* 34, 306–315 (2019).
19. Gray, W. R. *et al*. The effects of temperature, salinity, and the carbonate system on Mg/Ca in Globigerinoides ruber (white): A global sediment trap calibration. *Earth Planet. Sci. Let.* 482, 607–620 (2018a).
20. Foster, G. L. *et al.* Interlaboratory comparison of boron isotope analyses of boric acid, seawater and marine CaCO_3_ by MC-ICPMS and NTIMS. *Chem. Geol.* 358, 1–14 (2013).
21. Ni, Y., Foster, G. L. & Elliott, T. The accuracy of δ^11^B measurements of foraminifers. *Chem. Geol.* 274:187–195 (2010).
22. Farmer, J. R., Hönisch, B. & Uchikawa, J. Single laboratory comparison of MC-ICP-MS and N-TIMS boron isotope analyses in marine carbonates. *Chem. Geol.* 447, 173–182 (2016).
23. Lee, K. *et al.* Global relationships of total alkalinity with salinity and temperature in surface waters of the world’s oceans, *Geophys. Res. Let.* 33, L19605, doi:10.1029/2006GL027207 (2006).
24. Dyez, K. A., Hönisch, B., & Schmidt, G. A. Early Pleistocene obliquity-scale *p*CO_2_ variability at ~1.5 million years ago. *Paleoceanogr. Paleoclimatol.* 33, 1270–1291 (2018).
25. Martinez-Boti, M. A. et al. Boron isotope evidence for oceanic carbon dioxide leakage during the last deglaciation. *Nature* 518, 219–222 (2015).
26. Gray, W. R. *et al.* Deglacial upwelling, productivity and CO_2_ outgassing in the North Pacific Ocean. *Nat. Geosci.* 11, 340–344 (2018).
27. Chalk, T. B. *et al*. Causes of ice age intensification across the mid-Pleistocene transition. *Proc. Natl Acad. Sci. USA* 114, 13114–13119 (2017).
28. Fraser, N. et al. Precipitation varia- bility within the West Pacific Warm Pool over the past 120 ka: Evidence from the Davao Gulf, southern Philippines, *Paleoceanogr*aphy 29, 1094–1110 (2014).
29. Partin, J. W., Cobb, K. M., Adkins, J. F., Clark, B. & Fernandez, D. P. Millennial- scale trends in west Pacific warm pool hydrology since the Last Glacial Maximum. *Nature* 449, 452–455 (2007).
30. Tachikawa, K., Timmermann, A., Vidal, L., Sonzogni, C. & Timm, O. E. CO_2_ radiative forcing and Intertropical Convergence Zone influences on western Pacific warm pool climate over the past 400 ka, *Quat. Sci. Rev.* 86, 24–34 (2014).
31. Lisiecki, L. E. & Raymo, M. E. Diachronous benthic δ^18^O responses during late Pleistocene terminations. *Paleoceanography* 24, PA3210 (2009).
32. Anand, P., Elderfield, H. & Conte, M. H. Calibration of Mg/Ca thermometry in planktonic foraminifera from a sediment trap time series. *Paleoceanography* 18, 1050 (2003).
33. Marcott, S. A. *et al.* Centennial-scale changes in the global carbon cycle during the last deglaciation. *Nature* 514, 616–619 (2014).
34. Ahn, J. & Brook, E. J. Siple Dome ice reveals two modes of millennial CO_2_ change during the last ice age. *Nat. Commun.*, doi:10.1038/ncomms4723 (2014).
35. Lüthi, D. *et al.* High-resolution carbon dioxide concentration record 650,000–800,000 years before present. *Nature* 453, 379–382 (2008).
